# The Global Burden of Type 2 Diabetes Attributable to Dietary Risks: Insights from the Global Burden of Disease Study 2019

**DOI:** 10.3390/nu15214613

**Published:** 2023-10-30

**Authors:** Alina Ioana Forray, Mădălina Adina Coman, Ruxandra Simonescu-Colan, Andreea Isabela Mazga, Răzvan Mircea Cherecheș, Cristina Maria Borzan

**Affiliations:** 1 Discipline of Public Health and Management, Department of Community Medicine, Iuliu Hațieganu University of Medicine and Pharmacy, Victor Babeș 8, 400347 Cluj-Napoca, Romania; 2 Department of Public Health, College of Political, Administrative and Communication Sciences, Babeș-Bolyai University, General Traian Moșoiu 71, 400132 Cluj-Napoca, Romaniaruxandra.simonescu@gmail.com (R.S.-C.);; 3 Faculty of General Medicine, Iuliu Hatieganu University of Medicine and Pharmacy, 400012 Cluj-Napoca, Romania

**Keywords:** Type 2 Diabetes Mellitus, Global Burden of Disease Study, dietary risk factors, Age-Standardized Mortality Rate, Age-Standardized DALY Rate, Socio-Demographic Index

## Abstract

The Global Burden of Disease Study (GBD) 2019 reveals an increasing prevalence of Type 2 Diabetes Mellitus (T2DM) from 1990 to 2019. This study delves into the role of dietary risk factors across different demographic and socioeconomic groups. Utilizing data from the GBD 2019, it analyzes age-adjusted T2DM metrics—death counts, Disability-Adjusted Life Years (DALYs), and Age-Standardized Rates (ASRs)—stratified by age, sex, and region. The study employed Estimated Annual Percentage Changes (EAPCs) to track trends over time. The results show that in 2019, 26.07% of T2DM mortality and 27.08% of T2DM DALYs were attributable to poor diets, particularly those low in fruits and high in red and processed meats. There was a marked increase in both the death rate and DALY rate associated with dietary risks over this period, indicating the significant impact of dietary factors on the global T2DM landscape. Geographic variations in T2DM trends were significant, with regions like Southern Sub-Saharan Africa and Central Asia experiencing the most substantial increases in Age-Standardized Mortality Rate (ASMR) and Age-Standardized DALY Rate (ASDR). A positive correlation was noted between Socio-Demographic Index (SDI) and T2DM burden due to dietary risk factors. The study concludes that targeted public health initiatives promoting dietary changes could substantially reduce the global T2DM burden.

## 1. Introduction

In recent decades, the global health landscape has witnessed a significant surge in the prevalence of Type 2 Diabetes Mellitus (T2DM), a chronic condition that not only burdens individuals but also strains healthcare systems and economies worldwide. The International Diabetes Federation (IDF) reported a global diabetes prevalence of 10.5% in adults in 2021, with projections indicating a rise to encompass one in eight adults by 2045 [1]. This alarming trend underscores the urgency needed to address the escalating public health crisis, with a particular focus on T2DM, which accounts for over 90% of all diabetes cases [1].

The Global Burden of Disease Study (GBD) 2019 delineated the substantial increase in the age-standardized incidence and mortality rates of T2DM from 1990 to 2019, highlighting the pressing need for comprehensive strategies to curb this trend [2,3]. The GBD 2019 further highlighted the substantial burden of diabetes, attributing it as the eighth leading cause of death and disability globally, with a notable increase in the fraction of burden due to disability since 1990 [4,5]. This burden is exacerbated by complications such as kidney disease and vision impairment, underscoring the multifaceted impact of diabetes on individual health [6].

The geographical distribution of this burden varies significantly, with European Union countries witnessing a consistent rise in age-standardized prevalence rates of T2DM and regions such as the Middle East and North Africa experiencing an almost 86% increase in average prevalence rates from 1990 to 2019 [7,8]. This scenario is particularly grim in low- and middle-income countries, which have registered the highest growth rates, indicating a pressing need for targeted interventions in these regions [3,9].

A deeper analysis of the risk factors associated with T2DM reveals a strong correlation between high body mass index (BMI) and dietary risks, which accounted for a substantial fraction of global T2DM disability-adjusted life years (DALYs) in 2021 [4,5]. The role of dietary patterns in the onset and progression of T2DM has been substantiated by numerous studies, emphasizing the detrimental effects of high intake of added sugars, saturated and trans fats, and the protective effects of a diet rich in fruits, vegetables, whole grains, and dietary fiber [10,11,12]. Dietary choices emerge as a pivotal modifiable risk factor in this context, with changes in dietary patterns over the last three decades significantly influencing the global burden of T2DM. The adoption of high-fat and Western diets has been associated with an increased risk of T2DM. In contrast, balanced dietary patterns and the Mediterranean diet have demonstrated protective effects against the disease [13,14]. However, the specific burden of T2DM attributable to dietary risks remains largely unexplored, with previous analyses focusing on the incidence of T2DM [15], isolated dietary factors [16,17,18], or specific geographical regions and countries [19].

The escalating global burden of T2DM necessitates immediate and targeted policy responses, focusing on lifestyle interventions such as diet modification, physical activity, and smoking cessation, among others. The present study aims to offer a detailed depiction of the global T2DM landscape attributable to seven dietary risk factors, facilitating a deeper understanding of the role of dietary risks in terms of mortality and disability, considering different demographic and socioeconomic groups, and elucidating the trends over the past three decades. The study seeks to fill the existing gaps in the literature by providing a comprehensive analysis of the impact of dietary risks on T2DM, incorporating seven dietary risk factors across the 21 GBD regions, 5 SDI regions, and 204 countries and territories. By fostering a comprehensive understanding of the global T2DM burden and its dietary determinants, the study aims to pave the way for informed policy interventions, thereby contributing to international efforts to mitigate the impact of this chronic condition on public health.

## 2. Materials and Methods

### 2.1. Data Sources and Definitions

This epidemiological study employs retrospective data extracted from the Global Burden of Disease 2019 (GBD 2019) database hosted by the Institute for Health Metrics and Evaluation (IHME) [20]. The GBD 2019 study encompasses comprehensive demographic and epidemiological information from 204 countries and regions, spanning the years 1990 to 2019, which were derived from multiple sources, including censuses, household surveys, disease registries, and others [21]. The current study included yearly age-adjusted information on the burden of T2DM attributable to dietary risk factors in adults from 1990 to 2019, stratified by age, sex, and region. The data cover various demographic groups in 204 countries, 21 global burden of disease regions, and five Socio-Demographic Index regions. We provide a short overview of the estimates from GBD 2019 here, although other authors have more comprehensively discussed them in separate publications [5,21,22]. The study was designed in adherence to the Guidelines for Accurate and Transparent Health Estimates Reporting (GATHER) statement [23].

### 2.2. Estimation of T2DM Burden

Metrics of interest included Age-Standardized Mortality Rates (ASMRs) and age-standardized disability-adjusted life-year rates (ASDRs) expressed per 100,000 persons. The GBD 2019 employs Disability-Adjusted Life Years (DALYs) as a composite measure, integrating Years of Life Lost (YLLs) due to premature mortality and Years Lived with Disability (YLDs) [24]. The International Classification of Diseases, Tenth Edition (ICD-10), served as the coding guideline for T2DM. Comprehensive information on input sources and pertinent metadata can be accessed through the online data source platform http://ghdx.healthdata.org/gbd-2019/data-input-sources (accessed on 1 August 2023).

### 2.3. Estimation of Attributable Burden

The proportion of T2DM burden attributable to dietary risk factors was calculated based on Population Attributable Fractions (PAFs). PAFs were estimated by comparing the theoretical minimum risk exposure level with actual population exposure levels, assuming all other risk factors remained constant. The attributable burden was derived by multiplying relevant PAFs by the overall T2DM burden for each age, sex, location, and year group [21].

### 2.4. Selection of Dietary Risk Factors

GBD 2019 categorizes risk factors into three main categories: behavioral, environmental and occupational, and metabolic. These are further divided into sub-categories, individual risk factors, or clusters of risk factors [21]. For T2DM, data on seven specific dietary risk factors relevant to the outcomes of T2DM were gathered from the Global Health Data Exchange query tool: http://ghdx.healthdata.org/gbd-results-tool (accessed on 01 August 2023). These risk factors included a diet low in fruits, fiber, whole grains, nuts, and seeds and high in red meat, processed meat, and sugar-sweetened beverages (Supplemental Table S1). All of these dietary risk factors were sourced from a 24 h dietary recall survey, in which food and nutrient consumption was reported in grams per individual per day. 

### 2.5. Socio-Demographic Index

The GBD 2019 study employs the Socio-Demographic Index (SDI) as a composite measure of socioeconomic status that strongly correlates with health outcomes. This index is the geometric mean of three scaled metrics ranging from 0 to 1: fertility rates for those under 25, average educational attainment for individuals 15 and older, and per capita income adjusted for time. According to the GBD 2019 study, the SDIs were sorted into five categories: high SDI (greater than 0.81), high-middle SDI (0.70–0.81), middle SDI (0.61–0.69), low-middle SDI (0.46–0.60), and low SDI (less than 0.46).

### 2.6. Statistical Analysis

To measure the burden of T2DM linked to dietary risk factors, variables such as the count of deaths or DALYs and Age-Standardized Rates (ASRs) were utilized, all within a 95% Uncertainty Interval (UI). To gauge temporal changes from 1990 to 2019 in age-standardized T2DM mortality and DALY rates attributable to dietary factors, we employed Estimated Annual Percentage Changes (EAPCs). Regression was used to model the change in ASRs over time as follows: ASR = α + β × year + ε. The EAPC and its 95% confidence interval (CI) were obtained via the formula 100 × (exp(β) − 1), and a linear regression analysis was conducted to investigate the relationship between ASR, SDI values, and EAPC [25]. All statistical analyses were performed using RStudio Desktop for MacOS (Version 2023.06 “Mountain Hydrangea”), and data visualizations were created using ggplot2 and RColorBrewer packages.

## 3. Results

### 3.1. Global Trends in T2DM Burden Attributable to Dietary Risk Factors from 1990 to 2019

Our analysis of the GBD 2019 data revealed that in 2019, 26.07% of T2DM mortality (95% UI: 21.58, 30.27) and 27.08% of T2DM DALYs (95% UI: 22.55, 31.30) were attributable to dietary risk factors (Supplemental Table S2). This represents a decrease from 27.93% (95% UI: 23.36, 32.12) and 28.34% (95% UI: 23.79, 32.59), respectively, in 1990. The total number of T2DM deaths due to dietary risks significantly increased from 169,361.31 (95% UI: 139,706.63, 197,685.95) in 1990 to 383,869.54 (95% UI: 314,627.19, 450,007.43) in 2019, while the number of DALYs similarly increased from 7.22 million (95% UI: 5.60, 8.87) to 17.96 million (95% UI: 13.65, 22,63) over the same period.

**Figure 1:**
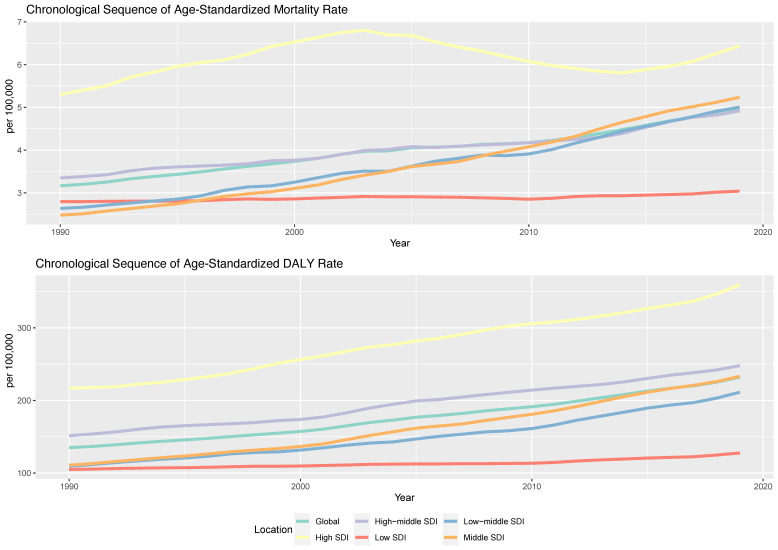
Age-standardized mortality and DALY rates for T2DM attributable to dietary risk factors from 1990 to 2019 by SDI regions.

The ASMRs and ASDRs of diet-related T2DM in 2019 were 4.96 (95% UI: 4.07, 5.82) per 100,000 and 232.12 (95% UI: 176.48, 292.50) per 100,000 people, respectively. The ASMRs (EAPC: 1.46; 95% CI:1.39, 1.52) and ASDRs (EAPC: 1.89; 95% CI: 1.85, 1.93) for T2DM attributable to dietary risk factors showed significant upward trends from 1990 to 2019 (Table 1, Figure 1). Our analysis identified three dietary risk factors that contributed the most to the ASMRs and ASDRs for T2DM from 1990 to 2019. These included a diet low in fruits, high in red meat, and high in processed meat (Table 1). 

Geographically, there was considerable heterogeneity in the trends of T2DM attributable to dietary risks. The largest annual increase of the ASMRs was observed in Southern Sub-Saharan Africa (+3.17%), Central Asia (+3.03%), followed by Andean Latin America (+2.67%) and South Asia (+2.61%), while ASDRs followed similar regional trends. Significant annual declines in ASMRs occurred in countries in Eastern, Central, and Western Sub-Saharan regions with a drop of 1.01%, 0.29%, and 0.16%, respectively, followed by Western Europe (−0.08%). Regarding the annual decrease in ASDRs, this was observed only in the Eastern Sub-Saharan African Region (−0.74%), while most of the regions had positive EAPCs, with Central Asia and Southern Sub-Saharan Africa having EAPCs larger than 3% (Table 2).

Regarding ASMR and ASDR trends for T2DM attributable to dietary risk factors of the five different SDI regions, most regions (Middle, Low-middle, and High-middle) displayed consistent upward trends. The Middle SDI region recorded the largest annual increase (EAPC in ASMR: 2.71%; EAPC in ASDR: 2.66%), while the Low-middle SDI region grew annually around 2.25% in ASMR and 2.22% in ASDR. However, the Low SDI region showed a significantly different trend, with a relatively stagnant annual growth of only about 0.23% in ASMR and 0.57% in ASDR. Despite fluctuating values over the years, the High SDI region had an overall increase of around 0.21% in ASMR and 1.77% in ASDR (Figure 1). 

**Figure 2:**
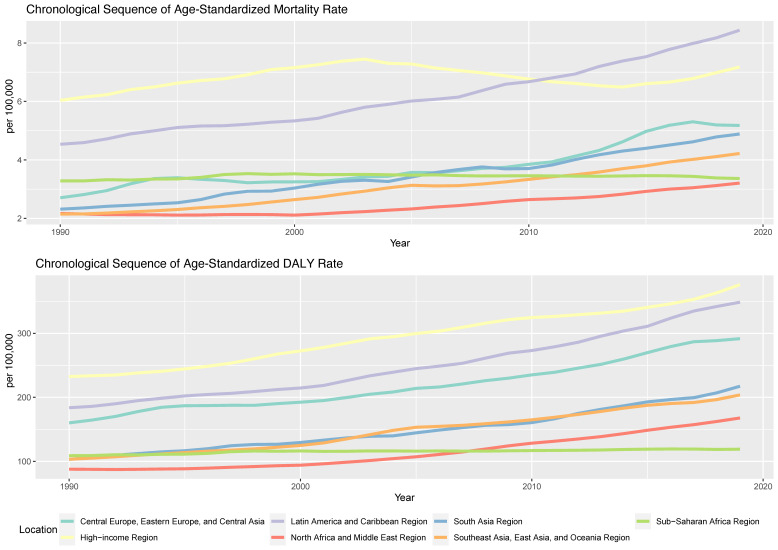
Age-standardized mortality and DALY rates for T2DM attributable to dietary risk factors from 1990 to 2019 by GBD super regions.

For the past three decades, the most noteworthy trends in ASMR across global regions have been observed in Southeast Asia, East Asia, Oceania, Latin America, and Caribbean regions. The high-income region showed the most fluctuations, and the North Africa and Middle East regions demonstrated the most modest increase. Regional trends in ASDR show the highest numerical value among all regions in the high-income region. The Asian regions (Southeast Asia, East Asia, Oceania, and South Asia) displayed the most rapid growth. In contrast, regions such as Sub-Saharan Africa and Central Europe, Eastern Europe, and Central Asia experienced slower growth rates or, in the latter case, a plateau (Figure 2).

**Figure 3:**
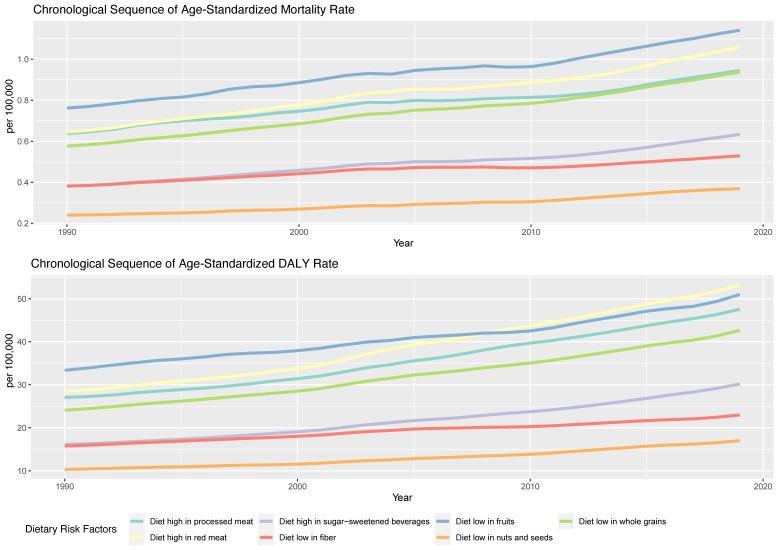
Age-standardized mortality and DALY rates for T2DM attributable to specific dietary risk factors from 1990 to 2019.

Regarding specific dietary factors (Figure 3, Table 1), an upward trend in the ASMRs and ASDRs of T2DM was observed for all dietary risk factors over the 30-year period. The risk factor associated with the highest ASMR was a diet low in fruits, with a rate of 1.14 per 100,000 in 2019 (EAPC: 1.3, 95% UI: 0.48, 1.0 6). Other significant annual increases were seen for a diet high in SSBs (EAPC: 1.63, 95% UI: 1.55, 1.71), a diet low in whole grains (EAPC: 1.62, 95% UI: 1.56, 1.67), and a diet high in red meat (EAPC: 1.61, 95% UI: 1.53, 1.6 8). Diets low in nuts and seeds showed a comparatively lower increase in death rates, though still notable at an EAPC of 1.5 1%. Regarding the ASDR of T2DM, the same upward trend was observed for all dietary risk factors, although the highest ASDR in 2019 was attributed to a diet high in red meat, with a rate of 53.29 per 100,000, which was followed by a diet low in fruits with a rate of 51.00 per 100,0 00. The highest annual increase in the DALY rate can be attributed to a diet high in red meat (EAPC: 2.28, 95% UI: 2.22, 2.34), a diet high in SSBs (EAPC: 2.18%, 95% UI: 2.12, 2.24), and a diet high in processed meat (EAPC: 2.06%, 95% UI: 2.01, 2.12). 

### 3.2. Global Trends in T2DM Attributable to Dietary Risk Factors by Gender and Age in 2019

**Figure 4:**
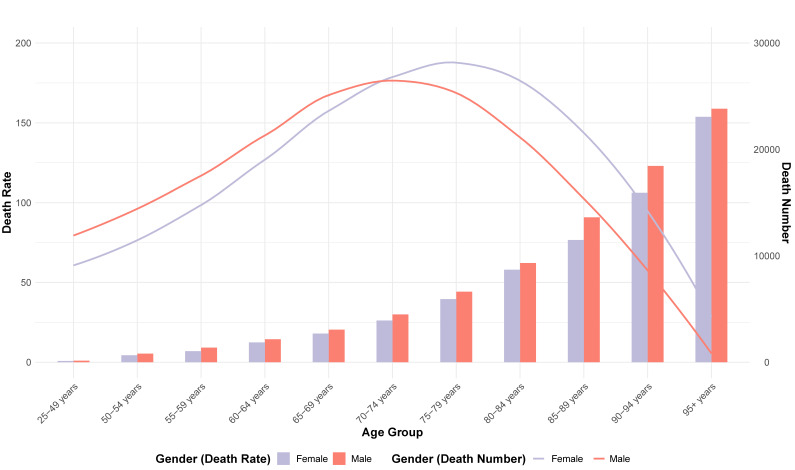
Age-Standardized Mortality Rate (bars) and death number (line) for T2DM attributable to dietary risk factors in 2019 by age and sex.

In 2019, the number of T2DM deaths attributable to dietary risk factors varied by age group, with a peak in women aged 75 to 84 and in men aged 70 to 74. In 2019, 197 thousand (95% UI: 161,507; 233,232) deaths were reported in women, and 186 thousand (95% UI = 152,303; 218,425) deaths were attributable to dietary risk factors. The T2DM mortality rate attributable to dietary risk factors was higher in men than women. In addition, the number of T2DM deaths was greater in men than in women under the age of 74 and greater in women than in men over 75. The T2DM mortality attributable to dietary risk factors increased with age in both sexes and was higher in men than in women (Figure 4). 

**Figure 5:**
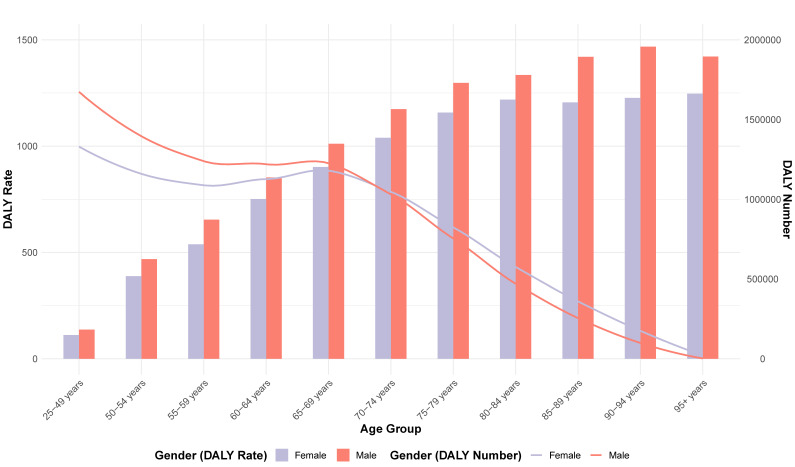
Age-Standardized DALY Rate per 100,000 people (bars) and DALYs number (line) for T2DM attributable to dietary risk factors in 2019 by age and sex.

In 2019, the peak number of DALYs due to T2DM attributable to dietary risk factors occurred in men and women aged 60 to 69. In addition, the number of DALYs due to T2DM attributable to dietary risk factors was higher in men than in women under the age of 74 and higher in women than in men over 75. The ASDR due to T2DM attributable to dietary risk factors was slightly higher in men than women and increased with age in both sexes (Figure 5). The DALYs were 8.744 million (95% UI = 6.702; 11.038) in females and 9.215 million (95% UI = 6.978; 11.589) in men. 

### 3.3. Global Trends in T2DM Burden Attributable to Dietary Risk Factors by Region

**Figure 6:**
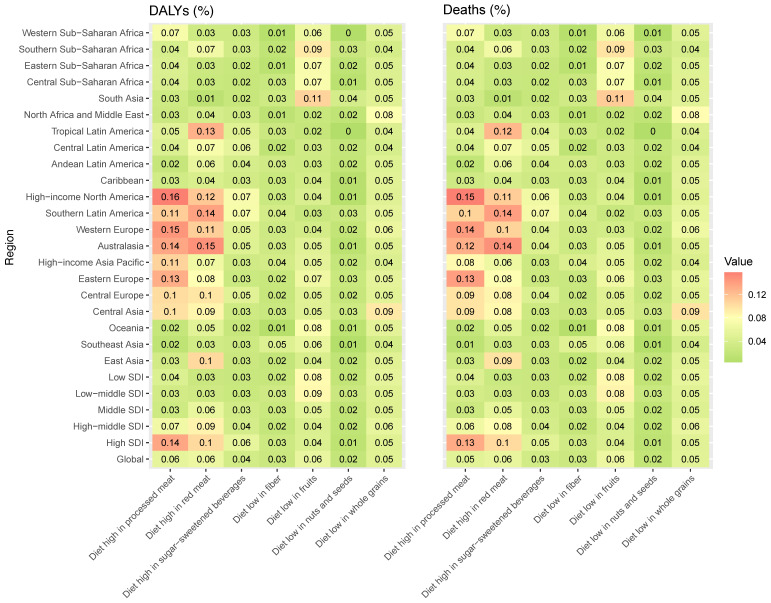
The proportion of deaths and DALYs for T2DM attributable to specific dietary risk factors in 2019.

Figure 6 presents the proportion of T2DM deaths and DALYs related to single dietary risks globally and for the 21 GBD and 5 SDI regions. The dietary risk “Diet high in processed meat” consistently exhibits high values in DALYs and deaths percentage in High-income North America, Western Europe, Australasia, Eastern Europe, Southern Latin America, High-Income Asia Pacific, Central Europe, Central Asia, Western Sub-Saharan Africa, and Tropical Latin Ameri ca. The dietary risk factor “Diet high in red meat” follows a similar trend with the highest DALYs and deaths percentage in Australasia, followed by Southern Latin America, Tropical Latin America, High-income North America, Western Europe, Central Europe, and East Asia. The regions with the highest values in the percentage share of DALYs and deaths for the dietary risk “Diet low in fruits” are South Asia, Southern Sub-Saharan Africa, Central Sub-Saharan Africa, Eastern Sub-Saharan Africa, and Western Sub-Saharan Africa. North Africa, the Middle East, and Central Asia have the highest DALYs and deaths percentage associated with a diet low in whole grains. Supplemental Tables S4 and S5 present the number of cases and ASR of deaths and DALYs attributable to diet-related T2DM in 204 countries and territories in 1990 and 2019 and the change in deaths and DALYs between 1990 and 2019.

In the High and High-middle SDI regions, there is a noticeable trend with higher values associated with diets high in processed meat and red meat. Specifically, in the High SDI region, the value for “Diet high in processed meat” is 0.14%, and for “Diet high in red meat”, it is 0.10%. Similarly, in the High-middle SDI region, the value for “Diet high in processed meat” is 0.07%, and for “Diet high in red meat,” it is 0.09%. Conversely, there is a distinct shift in dietary patterns when we examine the Low and Low-middle SDI regions. These regions exhibit higher values for diets low in fruits. For instance, in the Low SDI region, the value for “Diet low in fruits” is 0.08%, and in the Low-middle SDI region, the “Diet low in fruits” value stands at 0.09%. Figure 6 suggests that as the Socio-Demographic Index (SDI) decreases, there is a pronounced transition from diets rich in processed and red meats to those lacking in fruits and whole grains.

### 3.4. Influences of SDI Values on T2DM Burden Attributable to Dietary Risk Factors

**Figure 7:**
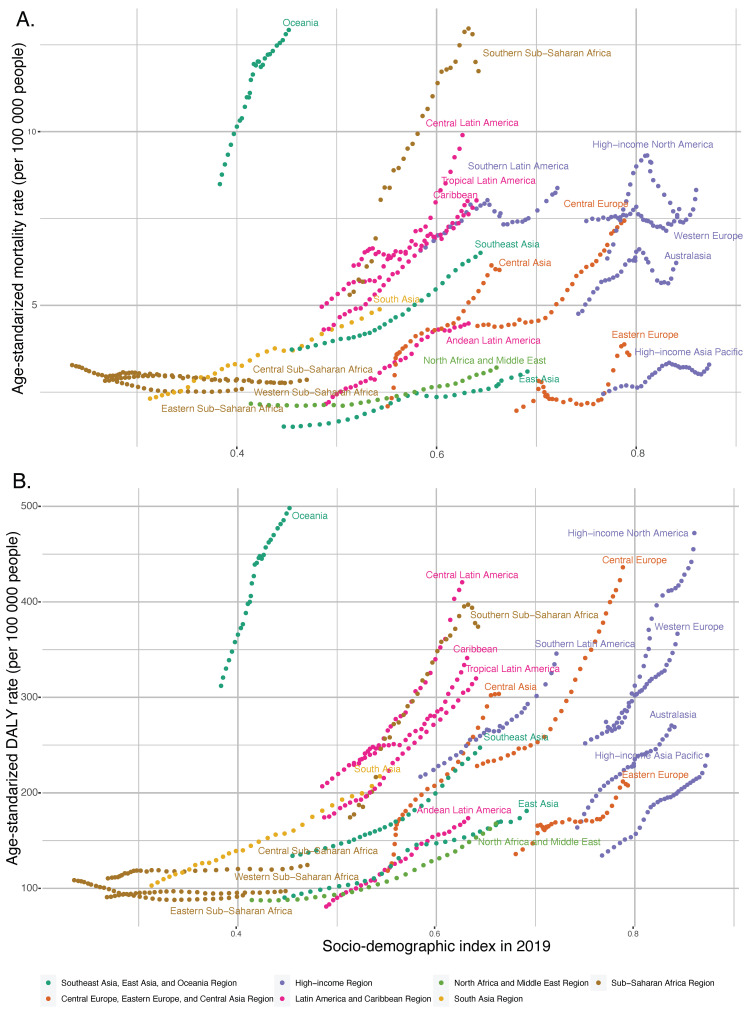
Age-standardized rates attributable to dietary factors across 21 Global Burden of Disease regions by Socio-Demographic Index, 1990–2019. (**A**) Mortality. (**B**) DALYs.

As the SDI value increased, the mortality and DALY rates for T2DM attributable to dietary risk factors gradually increased in most regions, with these rates increasing more slowly in low SDI regions than in high SDI regions. Central Europe, Oceania, Central Latin America, and Southern Sub-Saharan Africa showed significantly increasing mortality and DALY rates for T2DM attributable to dietary risk factors from 1990 to 2019. Interestingly, Oceania had the highest DALY and mortality rates for T2DM attributable to dietary risk factors, with an SDI value close to 0.452, a DALY rate of 498.11, and a mortality rate of 12.92 per 100,000 for T2DM attributable to dietary risk factors in 2019, which were significantly higher than in other regions with the same SDI values (Figure 7).

**Figure 8:**
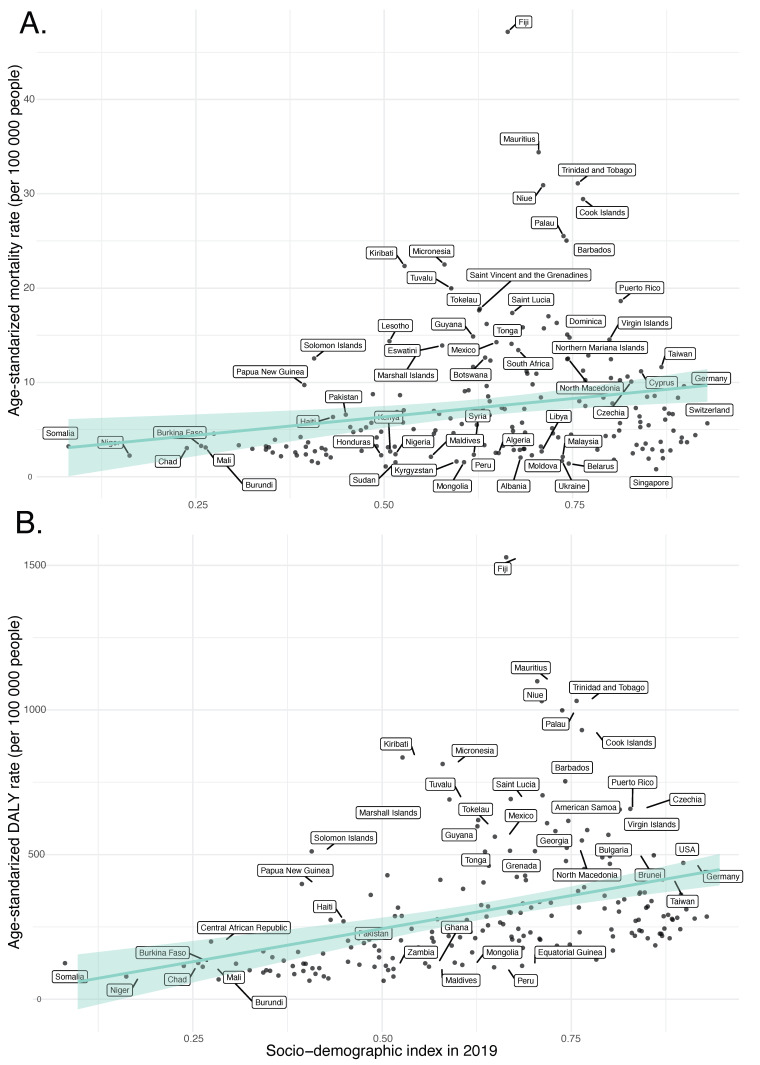
Age-standardized rates attributable to dietary factors across 204 countries and territories by Socio-Demographic Index in 2019. (**A**) Mortality, (**B**) DALYs.

In our regression analyses (Figure 8, Figures S1 and S3), the SDI in 2019 is a statistically significant predictor for the ASMR and ASDR of diet-related T2DM across 204 countries. Specifically, the model for ASMR indicated a significant positive relationship with the SDI value (β = 7.781, *p* = 0.003), accounting for approximately 4.2% of the variance in ASMR (Adjusted R-squared = 0.03 7). Similarly, the ASDR model revealed a substantial positive association with the SDI value (β = 457.58, *p* < 0.001), explaining around 13.4% of the variance in ASDR (Adjusted R-squared = 0.129). 

**Figure 9:**
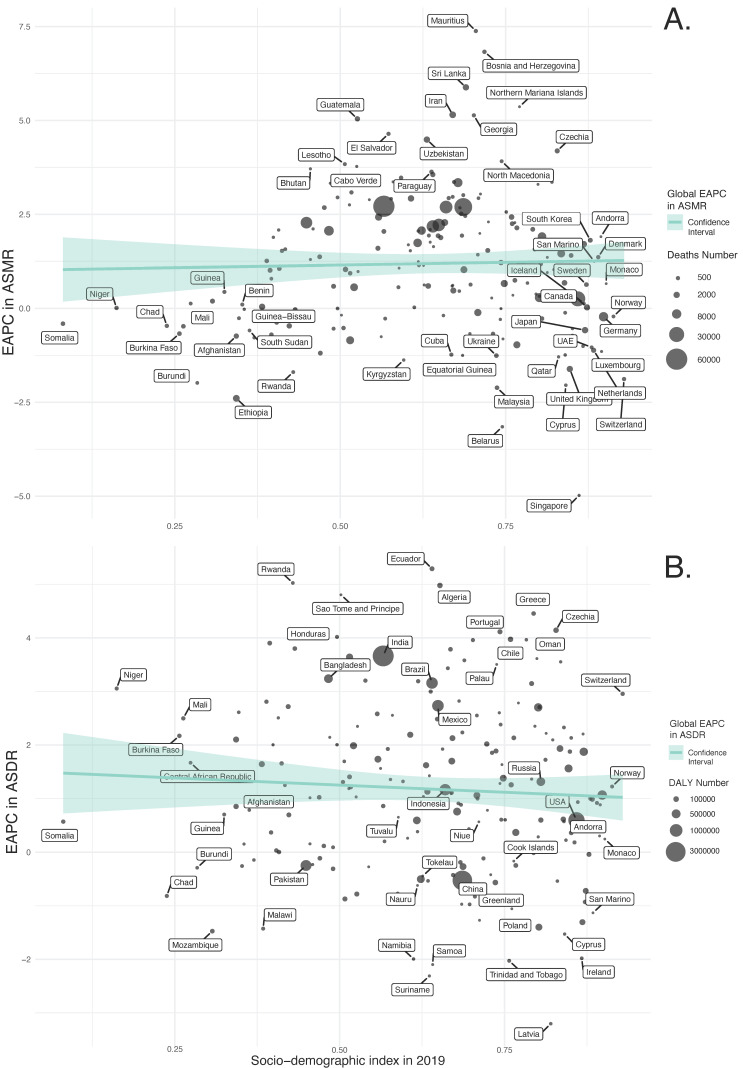
EAPCs in age-standardized rates attributable to dietary factors across 204 countries and territories by SDI in 2019. (**A**) Mortality. (**B**) DALYs.

The regression analyses indicate that the EAPCs in ASMR and ASDR have weak and non-statistically significant associations with the SDI values in 2019 (Figure 9, Figures S2 and S4); for ASMR, a slightly positive, non-significant trend was noted (β = 0.282, *p* = 0.703), whereas for ASDR, there was a minor yet non-significant negative relationship (β = −0.534, *p* = 0.411).

## 4. Discussion

Our analysis of the GBD data provides a comprehensive overview of the global burden of T2DM attributable to dietary risks from 1990 to 2019. The study delineates a significant increase in both T2DM mortality and DALYs over the three decades, with a notable contribution from dietary risk factors such as low fruit intake and high consumption of red and processed meats. The geographical heterogeneity in the trends of T2DM attributable to dietary risks is considerable, with regions such as Southern Sub-Saharan Africa and Central Asia witnessing the largest annual increase in ASMR and ASDR. Moreover, the data reveal a positive correlation between SDI values and the burden of T2DM attributable to dietary risk factors in most regions.

The upward trajectory in both T2DM mortality and DALYs over the past three decades can be attributed to a myriad of factors, including population aging and growth and increases in urbanization and modernization, which foster sedentary lifestyles and obesity [26]. Over the years, the shift toward unhealthy diets and reduced physical activity further aggravates the risks, calling for initiatives promoting healthy lifestyles [27]. The observation that mortality rates associated with T2DM fluctuated in high-income countries over the study period stands in stark contrast to the rapid increasing mortality rates witnessed in other regions, especially low- and middle-income countries. The observed fluctuations and slower growth in diabetes-related ASMR in high-income countries can be ascribed to a confluence of clinical, lifestyle, and systemic factors. Clinical advancements, which have precipitated a substantial decline in vascular disease mortality rates, translate to a shift in the cause of death, moving from vascular complications to cancers as the leading contributor to diabetes-related deaths [28]. Concurrently, lifestyle modifications in these populations have been instrumental in lowering cardiovascular complications related to diabetes mortality [29]. These trends are distinctively disparate from low- and middle-income countries, where an escalation in risk factors such as diet, obesity and smoking has been observed [29].

Our analysis identified three dietary risk factors that contributed the most to the ASMR and ASDR for T2DM from 1990 to 2019: a diet low in fruits, a diet high in red meat, and a diet high in processed meat. Certain fruits, being a rich source of fiber, vitamins, minerals, antioxidants, and polyphenols, can help regulate the levels of blood sugar and improve insulin sensitivity [30,31,32]. Conversely, meat, especially processed red meat, has been associated with an increased risk of T2DM due to its high levels of saturated fat, heme iron, and other compounds that can contribute to insulin resistance and inflammation. Processed meats, often high in sodium, nitrates, and other additives, can negatively affect blood sugar levels, chronic inflammation, oxidative stress, and insulin resistance [33,34,35]. In conclusion, these dietary factors may have a more significant impact on T2DM burden compared to other dietary risk factors due to their direct influence on blood sugar levels, insulin sensitivity, and inflammation. Addressing diet quality through initiatives in public health, clinical settings, and policy can help reduce the burden of T2DM mortality and DALYs globally.

The gender and age distribution of T2DM deaths attributable to dietary risk factors in 2019 reveals a higher burden in older adults, peaking in women aged 75 to 84 and in men aged 70 to 74. The peak in T2DM deaths for women and men occurs in the older age groups, aligning with global and regional trends that indicate increased mortality and morbidity of T2DM with advancing age, as rates of hypoglycemia and microvascular complication increase dramatically with long-term diabetes [36,37,38]. Our results show greater ASMRs in women than men after the age of 74, while ASDRs are consistently higher in men than women, which can be explained by both biological and psychosocial factors [39]. These differences in mortality rates can be influenced by genetic factors, lifestyle, and environmental factors that vary between males and females [40]. Sex hormones also play a significant role in glycemic status, body composition, vascular function, and inflammatory responses, which can impact the development and progression of T2DM and cardiovascular complications associated with diabetes [41,42]. Vandenheede et al. found that educational inequalities in diabetes mortality exist in both genders across Europe, but relative inequalities are generally more pronounced among women [43].

Our regression analyses indicate that the SDI in 2019 is a statistically significant predictor for ASMR and ASDR of diet-related T2DM across 204 countries, but no significant association was identified with the annual change in ASMR or ASDR. Although it has been reported that the burden of T2DM decreases with increasing SDI [44,45], diet-related factors contribute to the burden of T2DM in higher SDI regions more than in lower SDI regions. This can be explained by the fact that high SDI regions are typically characterized by the higher availability of unhealthy food options and more sedentary lifestyles, which contribute to the increased burden of T2DM [46]. Taking into consideration the lack of association with the annual percentage change in ASMRs and ASDRs, our study indicates that the ASRs of mortality and disability caused by diet-attributable T2DM exhibit a similar upward trend worldwide in all SDI regions. 

### Limitations

Despite the valuable insights provided by the GBD 2019 data, they share the limitations inherent to the GBD data. The modeling approach used estimates risk based on available dietary risk data but does not establish causation and should be regarded as approximations of risk. Insufficient data in many locations worldwide results in less accurate results. To mitigate the impact of data scarcity, we focus on regions instead of individual countries. When comparing data across countries, caution is advised. The study also faces challenges in accurately estimating the burden of T2DM due to inconsistencies in data sources, limited data on diabetes type, and variability in risk factors across countries and income tiers [2,3,22]. The adoption of a uniform standard for evaluating the burden of disease in the GBD database is complicated by the variable pace of economic development and the diverse foundations of health systems across countries. The creation of national or subnational diabetes registries can offer an exhaustive epidemiological landscape, enriching our understanding of diabetes risk factors and outcomes on both individual and aggregate levels [2]. Furthermore, the exclusion of kidney disease as a non-fatal sequela of diabetes from the GBD methodological framework means that the diabetes-associated years lived with disability reported are an underestimate [4]. Our analysis further disaggregated the data by variables such as age, gender, SDI, and geographical regions; however, the lack of comprehensive, reliable data on other social determinants of health poses a gap in our understanding of the global disparities in T2DM associated with dietary factors. Additionally, the study did not encompass other dietary factors that may exert an influence on T2DM, potentially resulting in a larger burden attributable to dietary factors. Finally, the Socio-Demographic Index (SDI) employed in the GBD 2019 is a regional-level metric that does not consider potential patient-level confounders, limiting its applicability in individual risk assessment. The GBD 2019 data provide valuable insights into public health and emphasizes the need for improved surveillance capabilities despite the limitations.

From a public health perspective, the increasing mortality and disability rates of T2DM attributable to dietary risk factors worldwide over the last three decades necessitate urgent interventions. Screening and education on diet can improve at-risk populations’ quality of life and reduce disease burden [47,48]. Public health campaigns targeting vulnerable groups, improved public health awareness regarding the link between T2DM and a diet high in red or processed meat or low in fruits and grains, and advocating for balanced diets can play a pivotal role in curbing the T2DM epidemic [49,50]. Moreover, policy interventions targeting food environments, such as taxing unhealthy foods and promoting healthy food options, can effectively reduce the burden of T2DM [51]. Addressing the multifaceted issue of the growing T2DM burden requires a holistic strategy encompassing targeted interventions to foster healthy lifestyles and control environmental risk factors, guided by insights from the recent data on global disease burden.

## 5. Conclusions

The increasing global burden of T2DM attributable to dietary risks underscores the urgent need for public health interventions targeting dietary quality. This study, leveraging the Global Burden of Disease 2019 data, offers a multidimensional and longitudinal analysis of T2DM across regions and countries attributable to seven key dietary risks. By identifying the most impactful dietary risk factors, our research provides actionable insights that could be a foundation for future research and policy formulation to mitigate the global T2DM burden. It is imperative to foster collaborative efforts across sectors and stakeholders to address the multifaceted challenges posed by T2DM, leveraging insights from the GBD 2019 data to guide targeted interventions and policy initiatives.

## Figures and Tables

**Table 1:** Global age-standardized rates and rates changes attributable to 7 dietary factors for T2DM burden, 1990 and 2019.

Dietary Factor	Age-Standardized Rate Per 100,000 People (95% UI)				Estimated Annual Percentage Change from 1990 to 2019 (95% CI)	
	1990		2019			
	Death Rate	DALY Rate	Death Rate	DALY Rate	Death Rate	DALY Rate
Dietary risks	3.17 (2.61, 3.70)	135.00 (104.85, 165.97)	4.96 (4.07, 5.82)	232.12 (176.48, 292.50)	1.46 (1.39, 1.52)	1.89 (1.85, 1.93)
Diet low in fruits	0.76 (0.48, 1.06)	33.36 (20.79, 48.12)	1.14 (0.72, 1.63)	51.00 (30.63, 76.36)	1.3 (1.24, 1.37)	1.35 (1.29, 1.40)
Diet high in red meat	0.65 (0.36, 0.90)	28.49 (16.32, 41.26)	1.06 (0.59, 1.49)	53.29 (31.46, 77.06)	1.61 (1.53, 1.68)	2.28 (2.22, 2.34)
Diet high in processed meat	0.64 (0.45, 0.77)	27.06 (18.61, 35.35)	0.95 (0.66, 1.14)	47.56 (31.42, 63.10)	1.2 (1.12, 1.28)	2.06 (2.01, 2.12)
Diet low in whole grains	0.58 (0.20, 0.85)	24.09 (8.43, 36.92)	0.94 (0.33, 1.40)	42.65 (14.60, 66.49)	1.62 (1.56, 1.67)	1.99 (1.95, 2.02)
Diet high in sugar-sweetened beverages	0.38 (0.26, 0.48)	16.15 (10.81, 21.30)	0.63 (0.39, 0.82)	30.17 (17.89, 41.87)	1.63 (1.55, 1.71)	2.18 (2.12, 2.24)
Diet low in fiber	0.38 (0.18, 0.59)	15.76 (7.11, 24.64)	0.53 (0.23, 0.84)	22.99 (9.39, 37.17)	1.03 (0.93, 1.12)	1.26 (1.21, 1.31)
Diet low in nuts and seeds	0.24 (0.06, 0.48)	10.30 (2.41, 20.66)	0.37 (0.11, 0.71)	17.01 (4.81, 33.20)	1.51 (1.44, 1.58)	1.76 (1.68, 1.84)

CI, confidence interval; DALY, disability-adjusted life year; UI, uncertainty interval.

**Table 2:** Age-standardized rates and estimated annual percentage change for diet-related T2DM by SDI and regions, 1990 and 2019.

Region	Age-Standardized Rate Per 100,000 People (95% UI)				Estimated Annual Percentage Change from 1990 to 2019 (95% CI)	
	1990		2019			
	Death Rate	DALY Rate	Death Rate	DALY Rate	Death Rate	DALY Rate
Global	3.17 (2.61, 3.70)	135.00 (104.85, 165.97)	4.96 (4.07, 5.82)	232.12 (176.48, 292.50)	1.46 (1.39, 1.52)	1.89 (1.85, 1.93)
SDI						
High SDI	5.30 (4.48, 6.10)	217.00 (170.58, 270.68)	6.44 (5.36, 7.49)	359.59 (267.05, 470.45)	0.21 (−0.07, 0.48)	1.77 (1.71, 1.83)
High-middle SDI	3.35 (2.80, 3.88)	151.44 (116.43, 189.58)	4.91 (4.04, 5.74)	247.97 (185.29, 318.94)	1.21 (1.14, 1.28)	1.73 (1.67, 1.78)
Middle SDI	2.48 (1.98, 2.96)	110.81 (85.27, 138.27)	5.23 (4.16, 6.21)	233.17 (178.27, 296.05)	2.71 (2.66, 2.76)	2.66 (2.6, 2.71)
Low-middle SDI	2.64 (2.10, 3.20)	109.27 (85.93, 134.46)	5.00 (4.08, 5.91)	211.27 (163.45, 261.36)	2.25 (2.18, 2.31)	2.22 (2.14, 2.3)
Low SDI	2.79 (2.21, 3.41)	104.85 (80.78, 128.68)	3.04 (2.42, 3.67)	127.66 (96.62, 158.69)	0.23 (0.19, 0.27)	0.57 (0.51, 0.63)
**Southeast Asia, East Asia, and Oceania Region**	2.15 (1.71, 2.58)	103.26 (77.86, 130.21)	4.22 (3.26, 5.11)	203.71 (153.06, 261.17)	2.45 (2.37, 2.53)	2.47 (2.38, 2.56)
East Asia	1.51 (1.18, 1.88)	90.39 (66.50, 118.01)	3.10 (2.35, 3.85)	181.14 (131.13, 241.61)	2.6 (2.39, 2.82)	2.59 (2.4, 2.78)
Southeast Asia	3.73 (2.90, 4.52)	134.13 (103.85, 164.34)	6.51 (4.89, 8.06)	247.24 (186.80, 309.68)	2.01 (1.86, 2.17)	2.11 (1.95, 2.27)
Oceania	8.49 (6.18, 11.04)	312.07 (222.80, 404.46)	12.93 (9.36, 17.15)	498.11 (353.54, 651.27)	1.3 (1.13, 1.46)	1.54 (1.41, 1.67)
**Central Europe, Eastern Europe, and Central Asia Region**	2.71 (2.30, 3.10)	160.13 (122.19, 203.10)	5.18 (4.26, 6.02)	291.70 (217.85, 377.29)	2.03 (1.74, 2.32)	1.98 (1.86, 2.09)
Central Asia	2.09 (1.77, 2.40)	118.48 (91.19, 149.12)	6.02 (4.91, 7.21)	303.60 (230.56, 383.03)	3.03 (2.69, 3.36)	3.03 (2.87, 3.19)
Central Europe	4.41 (3.67, 5.08)	228.09 (173.66, 288.47)	7.43 (6.00, 8.95)	436.28 (321.48, 571.21)	2.14 (1.95, 2.34)	2.48 (2.32, 2.65)
Eastern Europe	1.97 (1.68, 2.26)	135.98 (103.24, 173.47)	3.57 (2.88, 4.27)	207.74 (155.38, 267.95)	1.33 (0.64, 2.02)	1.08 (0.88, 1.28)
**High-income Region**	6.04 (5.12, 6.92)	232.48 (183.56, 288.60)	7.18 (6.01, 8.34)	376.21 (279.78, 490.85)	0.19 (−0.03, 0.42)	1.68 (1.61, 1.75)
High-income Asia Pacific	2.45 (1.99, 2.91)	134.45 (99.88, 173.86)	3.30 (2.59, 3.99)	239.35 (169.86, 320.99)	0.82 (0.56, 1.09)	1.73 (1.54, 1.92)
Australasia	4.76 (4.07, 5.44)	163.79 (131.31, 201.31)	6.22 (5.16, 7.28)	268.90 (201.59, 346.25)	0.57 (0.26, 0.88)	1.48 (1.33, 1.63)
Western Europe	7.43 (6.24, 8.53)	251.82 (198.36, 312.94)	7.77 (6.42, 9.10)	366.59 (268.99, 480.43)	−0.08 (−0.17, 0.01)	1.17 (1.12, 1.23)
Southern Latin America	6.57 (5.59, 7.48)	216.87 (174.98, 259.84)	8.38 (7.07, 9.63)	345.82 (266.94, 437.30)	0.45 (0.26, 0.65)	1.35 (1.23, 1.47)
High-income North America	6.35 (5.40, 7.30)	274.25 (218.41, 340.33)	8.32 (6.98, 9.64)	472.14 (365.09, 610.56)	0.24 (−0.21, 0.69)	2.18 (2.04, 2.32)
**Latin America and Caribbean Region**	4.54 (3.61, 5.42)	183.84 (141.85, 230.77)	8.44 (6.71, 10.07)	348.72 (266.30, 437.57)	2.08 (1.99, 2.17)	2.24 (2.15, 2.34)
Caribbean	6.14 (4.61, 7.65)	229.35 (169.25, 295.13)	8.00 (5.69, 10.21)	341.23 (243.42, 456.60)	0.72 (0.6, 0.85)	1.25 (1.13, 1.37)
Andean Latin America	2.16 (1.61, 2.70)	80.96 (59.43, 102.90)	4.48 (3.23, 5.79)	173.56 (126.22, 227.38)	2.67 (2.53, 2.81)	2.7 (2.61, 2.79)
Central Latin America	4.96 (3.93, 5.94)	206.80 (158.01, 261.31)	9.90 (7.67, 12.15)	420.57 (318.47, 537.62)	2.23 (2.04, 2.41)	2.42 (2.27, 2.58)
Tropical Latin America	4.30 (3.44, 5.13)	174.39 (135.64, 219.59)	8.02 (6.57, 9.50)	319.77 (248.35, 397.51)	2.21 (2.16, 2.27)	2.25 (2.18, 2.32)
**North Africa and Middle East Region**	2.17 (1.60, 2.76)	87.74 (63.62, 113.42)	3.21 (2.43, 4.01)	167.81 (118.61, 223.20)	1.51 (1.31, 1.72)	2.48 (2.27, 2.69)
**South Asia Region**	2.32 (1.83, 2.87)	102.98 (79.08, 129.34)	4.89 (3.98, 5.90)	217.29 (166.32, 271.73)	2.61 (2.52, 2.71)	2.46 (2.37, 2.55)
**Sub-Saharan Africa Region**	3.28 (2.61, 3.96)	108.87 (85.13, 131.84)	3.36 (2.66, 4.09)	118.99 (93.62, 145.19)	0.09 (0, 0.17)	0.28 (0.23, 0.33)
Central Sub-Saharan Africa	3.87 (3.08, 4.68)	112.10 (88.26, 136.49)	4.24 (3.38, 5.15)	130.10 (102.65, 158.47)	−0.29 (−0.38, −0.21)	0.31 (0.25, 0.36)
Eastern Sub-Saharan Africa	2.76 (2.20, 3.36)	109.63 (86.76, 133.08)	3.02 (2.39, 3.67)	118.39 (93.42, 144.10)	−1.01 (−1.15, −0.86)	−0.74 (−0.89, −0.6)
Southern Sub-Saharan Africa	2.42 (1.92, 2.95)	88.60 (69.88, 107.80)	2.51 (1.99, 3.07)	94.28 (74.34, 114.88)	3.17 (2.77, 3.57)	3.01 (2.71, 3.32)
Western Sub-Saharan Africa	4.11 (3.27, 5.02)	118.08 (93.62, 142.96)	4.47 (3.55, 5.45)	136.21 (108.19, 165.09)	−0.16 (−0.26, −0.07)	0.14 (0.08, 0.19)

CI, confidence interval; DALY, disability-adjusted life year; UI, uncertainty interval. Bold font denotes the seven Super Regions, which are further sub-divided into 21 GBD Regions.

## Data Availability

All data can be extracted from the Global Health Data Exchange (GHDx) website http://ghdx.healthdata.org/gbd-results-tool (accessed on 1 August 2023).

## Supplementary Materials

The following supporting information can be downloaded at: https://www.mdpi.com/article/10.3390/nu15214613/s1, Table S1: Dietary Risk factors, definitions, and summary exposure values (Source: Global burden of 87 risk factors in 204 countries and territories, 1990–2019: a systematic analysis for the Global Burden of Disease Study 2019); Table S2: Percentage of T2DM burden attributable to various risks in 2019; Table S3: The number of cases and ASR of deaths attributable to diet-related T2DM in 204 countries and territories in 1990 and 2019 and the change in deaths between 1990 and 2019; Table S4: The number of cases and ASR of DALYs attributable to diet-related T2DM in 204 countries and territories in 1990 and 2019 and the change in DALYs between 1990 and 2019; Figure S1: ASMR for T2DM attributable to dietary risk factors in 2019 in 204 countries and territories; Figure S2: EAPC of ASMR for T2DM attributable to dietary risk factors, between 1990 and 2019 in 204 countries and territories; Figure S3: ASDR for T2DM attributable to dietary risk factors in 2019 in 204 countries and territories; Figure S4: EAPC of ASDR for T2DM attributable to dietary risk factors, between 1990 and 2019 in 204 countries and territories.

## Abbreviations

ASDR: Age-standardized disability-adjusted life-year rate; ASMR: Age-Standardized Mortality Rate; ASR: Age-standardized rate; CI: Confidence interval; DALY: Disability-adjusted life year; DRF: dietary risk factors; EAPC: Estimated annual percentage change; GATHER: Guidelines for Accurate and Transparent Health Estimates Reporting; GBD: Global Burden of Disease Study; ICD-10: International Classification of Diseases, Tenth Edition; IDF: International Diabetes Federation; IHME: Institute for Health Metrics and Evaluation; PAFs: Population Attributable Fractions; SDI: Socio-Demographic Index; T2DM: Type 2 Diabetes Mellitus; UI: Uncertainty interval.
