# Supplementary material for: The Global Burden of Type 2 Diabetes Attributable to Dietary Risks: Insights from the Global Burden of Disease Study 2019
Source: Nutrients. 2023 Oct 30;15(21):4613. doi: 10.3390/nu15214613 (PMC10648266; doi:10.3390/nu15214613)
Supplement: Supplementary file 1 [file nutrients-15-04613-s001.zip › nutrients-2671025-supplementary.pdf]

Supplementary Information

The online version contains supplementary material available at [insert link].

Table S1. Dietary Risk factors, definitions, and summary exposure values (Source: Global burden of 87 risk factors in 204 countries and territories, 1990–2019: a systematic analysis for the Global Burden of Disease Study 2019)

Table S2. Percent of T2DM burden attributable to various risks in 2019

Table S3. The number of cases and ASR of deaths attributable to diet-related T2DM in 204 countries and territories in 1990 and 2019 and the change of deaths between 1990 and 2019

Table S4. The number of cases and ASR of DALYs attributable to diet-related T2DM in 204 countries and territories in 1990 and 2019 and the change of DALYs between 1990 and 2019

Figure S1. ASMR for T2DM attributable to dietary risk factors in 2019 in 204 countries and territories

Figure S2. EAPC of ASMR for T2DM attributable to dietary risk factors, between 1990 and 2019 in 204 countries and territories

Figure S3. ASDR for T2DM attributable to dietary risk factors in 2019 in 204 countries and territories

Figure S4. EAPC of ASDR for T2DM attributable to dietary risk factors, between 1990 and 2019 in 204 countries and territories

Table S1. Dietary Risk factors, definitions, and summary exposure values (Source: Global burden of 87 risk factors in 204 countries and territories, 1990–2019: a systematic analysis for the Global Burden of Disease Study 2019)

**Risk factors**	**Definition**	**Summary exposure values 2019**
Diet low in fruit	Average daily consumption of fruit should be no less than 310-340 grams per day, including fresh, frozen, cooked, canned, or dried fruit, but excluding fruit juices and salted or pickled fruits.	56.86 (49.36, 65.37)
Diet low in fiber	Average daily fiber consumption from fruits, vegetables, grains, legumes, and pulses should be at least 21-22 grams daily.	27.62 (18.60, 36.95)
Diet low in nuts and seeds	Average daily nut and seed consumption, including tree nuts and peanuts, should be at least 10-19 grams per day.	47.47 (23.73, 66.35)
Diet low in whole grains	Average daily consumption of 140-160 grams of whole grains per day from various sources like breakfast cereals, bread, rice, pasta, biscuits, muffins, tortillas, pancakes, and others.	78.81 (71.06, 86.78)
Diet high in red meat	Red meat intake, excluding poultry, fish, eggs, and processed meats, in grams per day.	43.94 (38.03, 49.58)
Diet high in processed meat	Consuming any amount of smoked, cured, salted, or chemically preserved meat daily	29.81 (19.04, 43.32)
Diet high in sugar-sweetened beverages (SSBs)	Intake of beverages with ≥50 kcal per 226.8 gram serving, including carbonated beverages, sodas, energy drinks, fruit drinks, excluding 100% fruit and vegetable juices.	30.36 (22.71, 43.05)

Table S2. Percent of T2DM burden attributable to various risks in 2019 (Source: Institute for Health Metrics and Evaluation (IHME). **GBD Results**. Seattle, WA: IHME, University of Washington, 2020. Available from https://vizhub.healthdata.org/gbd-results/)

Measure	Risks	Percent (%)	95% UI	
			Lower	Upper
Deaths	1. Environmental/occupational risks	25,64	20,11	31,51
	1.1. Particulate matter pollution	19,86	14,20	25,49
	1.1.1. Ambient particulate matter pollution	13,36	9,49	17,53
	1.1.2. Household air pollution from solid fuels	6,50	4,22	9,53
	1.2. Non-optimal temperature	7,17	4,64	10,20
	1.2.1. High temperature	2,15	0,44	5,31
	1.2.2 Low temperature	5,07	2,75	7,28
	2. Behavioral risks	43,08	37,12	48,78
	2.1. Tobacco	15,99	11,07	20,10
	2.1.1. Smoking	8,22	6,71	9,78
	2.1.2. Secondhand smoke	8,38	3,23	12,86
	2.2. Alcohol use	-0,21	-1,86	1,38
	2.3. Dietary risks	26,07	21,58	30,27
	2.3.1. Diet low in fruits	6,00	3,70	8,59
	2.3.2. Diet low in whole grains	4,93	1,69	7,38
	2.3.3. Diet low in nuts and seeds	1,94	0,56	3,69
	2.3.4. Diet high in red meat	5,57	3,17	7,83
	2.3.5. Diet high in processed meat	4,97	3,49	5,94
	2.3.6. Diet high in sugar-sweetened beverages	3,33	2,07	4,28
	2.3.7. Diet low in fiber	2,78	1,25	4,40
	2.4. Low physical activity	8,50	4,24	14,10
	3. Metabolic risks	100,00	100,00	100,00
	3.1. High fasting plasma glucose	100,00	100,00	100,00
	3.2. High body-mass index	42,06	30,19	54,39
DALYs	1. Environmental/occupational risks	22,15	16,42	27,75
	1.1. Particulate matter pollution	19,55	13,94	25,00

1.1.1. Ambient particulate matter pollution	13,63	9,73	17,87
1.1.2. Household air pollution from solid fuels	5,92	3,81	8,64
1.2. Non-optimal temperature	3,25	1,98	4,86
1.2.1. High temperature	1,07	0,21	2,68
1.2.2 Low temperature	2,19	1,10	3,37
2. Behavioral risks	44,11	38,35	49,66
2.1. Tobacco	17,87	12,76	22,12
2.1.1. Smoking	9,86	8,24	11,53
2.1.2. Secondhand smoke	8,75	3,40	13,39
2.2. Alcohol use	-0,07	-2,09	1,91
2.3. Dietary risks	27,08	22,55	31,30
2.3.1. Diet low in fruits	5,95	3,66	8,56
2.3.2. Diet low in whole grains	4,97	1,72	7,43
2.3.3. Diet low in nuts and seeds	1,99	0,59	3,79
2.3.4. Diet high in red meat	6,22	3,77	8,55
2.3.5. Diet high in processed meat	5,55	3,90	6,70
2.3.6. Diet high in sugar-sweetened beverages	3,52	2,12	4,59
2.3.7. Diet low in fiber	2,68	1,16	4,27
2.4. Low physical activity	6,87	3,32	11,88
3. Metabolic risks	100,00	100,00	100,00
3.1. High fasting plasma glucose	100,00	100,00	100,00
3.2. High body-mass index	51,90	38,86	64,46

DALY, disability-adjusted life-years; UI, uncertainty interval.

Table S3. The number of cases and ASR of deaths attributable to diet-related T2DM in 204 countries and territories in 1990 and 2019 and the change of deaths between 1990 and 2019 (Source: Institute for Health Metrics and Evaluation (IHME). **GBD Results**. Seattle, WA: IHME, University of Washington, 2020. Available from https://vizhub.healthdata.org/gbd-results/)

**Country**	**1990**		**2019**		**1990-2019**
	**Deaths cases**	**ASMR per 100,000**	**Deaths cases**	**ASMR per 100,000**	**EAPC in ASMR**
	**No. (95% *UI*)**	**No. (95% *UI*)**	**No. (95% *UI*)**	**No. (95% *UI*)**	**No. (95% *CI*)**
Afghanistan	541,19 (333,67, 854,48)	4,74 (2,92, 7,48)	1238,44 (698,02, 1909,92)	3,24 (1,82, 4,99)	-0,74 (-1,05, -0,42)
Albania	28,55 (23,18, 34,19)	0,86 (0,7, 1,03)	55,29 (39,16, 74,35)	2,03 (1,44, 2,73)	2,67 (2,19, 3,16)
Algeria	392,73 (257,64, 577,44)	1,55 (1,02, 2,28)	1051,01 (708,65, 1452,54)	2,51 (1,69, 3,47)	1,88 (1,53, 2,23)
American Samoa	3,24 (2,32, 4,21)	6,69 (4,78, 8,69)	8,73 (6,3, 11,27)	15,74 (11,35, 20,3)	3,04 (2,78, 3,3)
Andorra	1,18 (0,84, 1,66)	2,18 (1,55, 3,07)	3,06 (2,3, 4,01)	3,68 (2,77, 4,83)	1,9 (1,75, 2,06)
Angola	367,88 (265,93, 493,02)	3,57 (2,58, 4,78)	826,92 (577,58, 1132,78)	2,74 (1,92, 3,76)	-1,19 (-1,35, -1,03)
Antigua and Barbuda	8,15 (6,36, 9,87)	13,41 (10,47, 16,26)	13,34 (10,31, 16,97)	15,08 (11,65, 19,18)	-0,01 (-0,31, 0,29)
Argentina	2469,88 (2103,34, 2833,36)	7,46 (6,35, 8,55)	3795,44 (3169,45, 4410,17)	8,41 (7,03, 9,78)	-0,11 (-0,38, 0,15)
Armenia	138,49 (112,79, 163,02)	4,06 (3,3, 4,77)	337,4 (251,3, 420,83)	11,17 (8,32, 13,94)	2,46 (1,58, 3,34)
Australia	821,63 (701,19, 938,73)	4,87 (4,16, 5,57)	1571,03 (1291,07, 1849,99)	6,39 (5,26, 7,53)	0,68 (0,37, 0,99)
Austria	514,07 (428,08, 597,62)	6,62 (5,51, 7,69)	755,98 (611,22, 903,24)	8,48 (6,86, 10,13)	1,4 (0,95, 1,86)
Azerbaijan	161,79 (128,94, 194,48)	2,21 (1,76, 2,65)	589,1 (443,45, 753,58)	5,73 (4,31, 7,33)	2,51 (2,07, 2,96)
Bahamas	18,57 (14,57, 22,54)	7,24 (5,68, 8,79)	35,97 (26,79, 46,57)	9,54 (7,11, 12,35)	0,38 (0,13, 0,63)
Bahrain	19,21 (13,81, 25,23)	3,78 (2,72, 4,97)	135,62 (90,48, 185,26)	9,4 (6,27, 12,84)	2,56 (2,28, 2,84)
Bangladesh	3262,06 (2622,53, 3996,61)	2,99 (2,4, 3,66)	9107,54 (6906,1, 11719,92)	5,72 (4,34, 7,36)	2,06 (1,75, 2,38)
Barbados	57,99 (46,63, 69,51)	22,84 (18,37, 27,38)	74,53 (54,61, 94,58)	25,03 (18,34, 31,76)	-0,29 (-0,52, -0,07)
Belarus	220,36 (188,03, 250,7)	2,1 (1,8, 2,39)	132,54 (100,85, 169,65)	1,4 (1,06, 1,79)	-3,15 (-3,73, -2,57)
Belgium	627,12 (519,88, 730,31)	6,28 (5,21, 7,32)	623,19 (499,12, 740,36)	5,46 (4,37, 6,48)	-0,74 (-0,91, -0,57)
Belize	6,24 (4,38, 8,08)	3,36 (2,36, 4,35)	22,93 (15,69, 30,07)	5,59 (3,83, 7,33)	1,69 (1,23, 2,14)
Benin	119,62 (92,95, 150,83)	2,47 (1,92, 3,11)	324,87 (235,59, 430,21)	2,56 (1,86, 3,4)	0,1 (-0,06, 0,26)
Bermuda	4,44 (3,43, 5,44)	7,47 (5,77, 9,15)	6,55 (5,03, 8,37)	10,23 (7,86, 13,08)	0,95 (0,81, 1,1)
Bhutan	10,67 (7,23, 14,67)	1,74 (1,18, 2,4)	39,73 (28,06, 53,6)	5,27 (3,72, 7,11)	3,71 (3,63, 3,79)
Bolivia	260,79 (184,08, 345,93)	4,06 (2,87, 5,39)	837,98 (589,62, 1138,18)	6,98 (4,91, 9,48)	2,04 (1,98, 2,11)

Bosnia and Herzegovina	136,14 (106,27, 164,44)	3 (2,34, 3,62)	561,71 (375,61, 755,49)	17,02 (11,38, 22,89)	6,83 (6,24, 7,42)
Botswana	84,33 (60,74, 112,42)	6,48 (4,67, 8,64)	295,47 (202,26, 405,96)	12,63 (8,65, 17,36)	1,93 (1,56, 2,31)
Brazil	6455,39 (5154,74, 7700,53)	4,34 (3,46, 5,17)	17339,6 (14209,5, 20568,95)	8 (6,56, 9,49)	2,18 (2,13, 2,23)
Brunei	23,55 (18,15, 29,67)	9,11 (7,02, 11,48)	46,45 (36,3, 56,03)	10,63 (8,3, 12,82)	1,03 (0,72, 1,35)
Bulgaria	649,71 (539,32, 756,84)	7,48 (6,21, 8,72)	780,13 (598,12, 986,48)	11,25 (8,63, 14,23)	0,75 (0,4, 1,1)
Burkina Faso	354,68 (272,78, 450,91)	3,71 (2,85, 4,72)	740,9 (567,63, 955,99)	3,27 (2,5, 4,21)	-0,67 (-0,83, -0,51)
Burundi	152,66 (100,48, 226,79)	2,74 (1,8, 4,07)	214,81 (140,43, 301,22)	1,8 (1,18, 2,52)	-1,98 (-2,21, -1,76)
Cambodia	5,43 (4,22, 7,39)	1,54 (1,2, 2,1)	32,43 (24,53, 40,23)	5,75 (4,35, 7,14)	3,78 (3,1, 4,45)
Cameroon	344,02 (246,39, 449,75)	3,32 (2,37, 4,33)	819,24 (593,58, 1070,96)	4,93 (3,58, 6,45)	1,37 (1,21, 1,52)
Canada	268,23 (185,34, 364,35)	2,58 (1,78, 3,51)	942,41 (650,8, 1331,13)	3,24 (2,24, 4,57)	0,59 (0,28, 0,9)
Cape Verde	1253,44 (1039,73, 1463,94)	4,6 (3,81, 5,37)	2211,09 (1811,29, 2622,74)	6,05 (4,96, 7,18)	0,03 (-0,64, 0,7)
Central African Republic	125 (92,11, 166,61)	4,56 (3,36, 6,07)	241,71 (167,64, 329,76)	4,56 (3,16, 6,22)	0,13 (0,08, 0,18)
Chad	201,47 (153,24, 260,95)	3,35 (2,54, 4,33)	497,62 (366,35, 659,41)	3,03 (2,23, 4,02)	-0,47 (-0,72, -0,21)
Chile	524,28 (442,08, 603,62)	3,95 (3,33, 4,55)	1459,22 (1209,53, 1707,27)	8,02 (6,65, 9,38)	2,43 (2,28, 2,57)
China	16985,75 (13217,48, 21229,31)	1,43 (1,12, 1,79)	41951,86 (31431,38, 52994,94)	2,95 (2,21, 3,73)	2,7 (2,48, 2,91)
Colombia	735,89 (559,13, 910,56)	2,26 (1,72, 2,8)	1601,05 (1126,33, 2172,63)	3,35 (2,36, 4,55)	0,6 (0,25, 0,94)
Comoros	12,86 (7,43, 18,47)	2,76 (1,6, 3,97)	29,25 (20,49, 39,97)	4,1 (2,87, 5,6)	1,3 (1,18, 1,42)
Congo	122,75 (88,92, 159,05)	5,02 (3,64, 6,51)	258,78 (183,49, 348,98)	4,91 (3,48, 6,63)	-0,27 (-0,45, -0,1)
Cook Islands	2,82 (2, 3,7)	14,81 (10,52, 19,45)	5,29 (3,74, 7,01)	29,43 (20,78, 38,97)	2,28 (2,14, 2,42)
Costa Rica	61,92 (45,89, 76,56)	2,04 (1,51, 2,52)	134,27 (92,99, 180,71)	2,85 (1,97, 3,83)	0,46 (-0,13, 1,05)
Cote d'Ivoire	224,16 (160,18, 293,21)	1,83 (1,31, 2,4)	706,58 (486,5, 931,93)	2,7 (1,86, 3,56)	1,06 (0,73, 1,38)
Croatia	177,86 (134,42, 219,2)	3,63 (2,74, 4,47)	275,45 (197,05, 364,58)	6,48 (4,64, 8,58)	1,57 (1,3, 1,83)
Cuba	505,36 (380,11, 628,49)	4,67 (3,51, 5,8)	401,15 (277,96, 534,96)	3,53 (2,45, 4,71)	-1,23 (-2,21, -0,24)
Cyprus	130,47 (96,68, 163,7)	16,77 (12,43, 21,04)	146,86 (111,76, 180,45)	11,18 (8,51, 13,74)	-2,05 (-2,26, -1,83)
Czech	561,64 (459,9, 655,77)	5,45 (4,47, 6,37)	1073,04 (835,23, 1347,38)	10,08 (7,85, 12,66)	4,19 (3,14, 5,25)
Denmark	429,29 (299,04, 575,16)	2,04 (1,42, 2,73)	873,38 (637,03, 1159,56)	3,33 (2,43, 4,42)	1,81 (1,72, 1,9)
Djibouti	929,85 (631,3, 1248,88)	2,41 (1,64, 3,24)	2262,02 (1577,44, 3077,22)	2,58 (1,8, 3,51)	0,04 (-0,12, 0,21)
Dominica	293,86 (246,61, 340,75)	5,71 (4,79, 6,62)	485,9 (396,21, 575,27)	8,37 (6,83, 9,91)	1,36 (0,91, 1,81)
Dominican Republic	11,53 (8,2, 15,75)	2,37 (1,69, 3,24)	56,97 (40,33, 79,79)	4,74 (3,35, 6,63)	2,11 (2,02, 2,2)
DR Congo	9,82 (7,25, 12,29)	13,27 (9,8, 16,6)	11,2 (8,03, 14,54)	16,31 (11,7, 21,17)	0,28 (0,07, 0,48)
Ecuador	137,09 (96,39, 175,16)	1,9 (1,34, 2,43)	503,09 (326,58, 707,54)	4,62 (3, 6,5)	3,47 (3,26, 3,68)
Egypt	232,91 (167,05, 295,83)	2,32 (1,67, 2,95)	1149,65 (788,13, 1536,83)	6,54 (4,48, 8,74)	3,56 (3,19, 3,92)

El Salvador	1062,31 (727,95, 1378,94)	1,91 (1,31, 2,48)	3263,66 (2093,59, 4784,58)	3,29 (2,11, 4,83)	2,28 (2,03, 2,53)
Equatorial Guinea	94,43 (67,27, 119,49)	1,79 (1,28, 2,27)	416,81 (279,23, 575,3)	6,66 (4,46, 9,2)	4,64 (4,31, 4,97)
Eritrea	18,56 (12,78, 25,3)	4,31 (2,97, 5,88)	41,77 (26,84, 62,42)	2,94 (1,89, 4,4)	-1,25 (-1,74, -0,76)
Estonia	74,46 (48,55, 110,05)	2,48 (1,62, 3,67)	213 (141,54, 301,09)	3,17 (2,11, 4,49)	0,79 (0,73, 0,85)
Eswatini	25,67 (20,85, 31,27)	1,64 (1,33, 1,99)	44,31 (31,96, 58,3)	3,38 (2,44, 4,44)	2,38 (1,44, 3,32)
Ethiopia	54,66 (38,81, 71,2)	6,78 (4,81, 8,83)	158,96 (106,2, 222,27)	13,92 (9,3, 19,46)	2,91 (2,32, 3,51)
Fiji	2714,99 (2044,96, 3639,36)	5,28 (3,98, 7,08)	3089,33 (2371,68, 3855,31)	2,87 (2,2, 3,58)	-2,4 (-2,66, -2,13)
Finland	141,46 (104,7, 185,78)	18,63 (13,79, 24,47)	429,89 (310,74, 563,02)	47,18 (34,1, 61,79)	2,93 (2,58, 3,28)
France	169,44 (141,11, 198,87)	3,38 (2,82, 3,97)	160,63 (129,96, 189,65)	2,9 (2,35, 3,43)	-0,92 (-1,18, -0,65)
Gabon	2709,97 (2271,64, 3137,15)	4,69 (3,93, 5,43)	4824,73 (3883,41, 5726,04)	7,29 (5,87, 8,65)	1,46 (0,94, 1,98)
Gambia	74,67 (52,77, 103,98)	7,53 (5,32, 10,49)	127,73 (88,34, 170,81)	7,3 (5,05, 9,76)	-0,19 (-0,27, -0,12)
Georgia	20,32 (14,61, 26,48)	2,05 (1,47, 2,67)	81,77 (58,88, 106,89)	3,64 (2,62, 4,76)	1,89 (1,58, 2,2)
Germany	181,87 (145,22, 220,76)	3,3 (2,64, 4,01)	400,36 (314,23, 500,37)	10,92 (8,57, 13,65)	5,13 (4,71, 5,57)
Ghana	7550,87 (6264,19, 8863,65)	9,45 (7,84, 11,09)	8156,15 (6671,92, 9652,65)	9,61 (7,86, 11,37)	-0,22 (-0,5, 0,06)
Greece	326,11 (234,41, 428,89)	2,17 (1,56, 2,86)	1037,53 (723,56, 1379,81)	3,29 (2,29, 4,38)	1,6 (1,32, 1,87)
Greenland	362,76 (303,59, 421,06)	3,49 (2,92, 4,05)	446,58 (367,89, 520,89)	4,32 (3,56, 5,04)	1,57 (1,05, 2,09)
Grenada	1,73 (1,38, 2,11)	3,11 (2,48, 3,8)	2,16 (1,64, 2,73)	3,85 (2,91, 4,86)	0,35 (0,19, 0,51)
Guam	10,38 (7,6, 13,09)	12,12 (8,87, 15,28)	14,55 (10,55, 18,54)	14,09 (10,22, 17,96)	0,59 (0,37, 0,81)
Guatemala	5,05 (3,7, 6,56)	3,69 (2,7, 4,79)	9,7 (6,99, 12,81)	5,68 (4,1, 7,51)	1,35 (1,06, 1,65)
Guinea	109,23 (80,49, 138,96)	1,37 (1,01, 1,74)	1253,45 (862,3, 1688,08)	7,05 (4,85, 9,5)	5,04 (4,41, 5,68)
Guinea-Bissau	165,73 (116,52, 219,65)	2,68 (1,88, 3,55)	373,8 (257,7, 514,25)	2,96 (2,04, 4,07)	0,44 (0,06, 0,82)
Guyana	39,28 (28,06, 52,36)	3,9 (2,79, 5,2)	74,64 (52,5, 102,53)	3,93 (2,76, 5,39)	-0,03 (-0,15, 0,1)
Haiti	62,17 (45,79, 78,52)	8,07 (5,95, 10,2)	114,62 (80,39, 151,67)	14,87 (10,43, 19,68)	2,06 (1,69, 2,42)
Honduras	411,03 (270,59, 570,72)	6,47 (4,26, 8,98)	785,48 (488,04, 1194,31)	6,33 (3,94, 9,63)	-0,03 (-0,09, 0,02)
Hungary	48,56 (33,8, 64,19)	1,03 (0,72, 1,36)	223,31 (156,43, 305,2)	2,28 (1,59, 3,11)	2,95 (2,78, 3,12)
Iceland	548,12 (449,6, 641,55)	5,27 (4,33, 6,17)	812,34 (623,05, 1021,99)	8,4 (6,44, 10,56)	2,1 (1,74, 2,46)
India	5,97 (5,09, 6,92)	2,35 (2, 2,73)	9,42 (7,56, 11,21)	2,73 (2,19, 3,25)	0,13 (-0,03, 0,29)
Indonesia	18042,21 (13751,38, 22855,51)	2,11 (1,61, 2,67)	63437,15 (49737,73, 78727,56)	4,56 (3,58, 5,66)	2,71 (2,59, 2,84)
Iran	6544,86 (5108,27, 8055,41)	3,53 (2,76, 4,35)	18613,74 (13224,77, 23609,36)	7,17 (5,1, 9,1)	2,69 (2,55, 2,84)
Iraq	524,92 (388,55, 667,25)	0,9 (0,66, 1,14)	2875,89 (2189,89, 3539,3)	3,41 (2,6, 4,2)	5,15 (4,81, 5,5)
Ireland	722,2 (495,66, 968,61)	4,1 (2,82, 5,5)	2039,34 (1449,73, 2658,39)	4,84 (3,44, 6,31)	0,47 (0,33, 0,62)

Israel	154,7 (130,14, 177,16)	4,3 (3,61, 4,92)	181,53 (149,82, 213,16)	3,7 (3,05, 4,34)	-1,01 (-1,17, -0,84)
Italy	197,83 (153, 237,03)	3,99 (3,08, 4,78)	723,23 (577,57, 872,07)	7,77 (6,2, 9,37)	1,18 (0,17, 2,2)
Jamaica	6274,16 (5297,45, 7143,48)	11,05 (9,33, 12,58)	7517,27 (6105,77, 8780,34)	12,46 (10,12, 14,56)	0,29 (0,19, 0,38)
Japan	203,59 (143,5, 260,96)	8,61 (6,07, 11,04)	445,23 (306,21, 587,79)	15,84 (10,89, 20,91)	1,93 (1,67, 2,2)
Jordan	2953,59 (2413,42, 3452,5)	2,35 (1,92, 2,74)	2501,98 (1917,47, 3045,66)	1,96 (1,5, 2,38)	-0,58 (-1, -0,16)
Kazakhstan	160,46 (117,36, 206,93)	4,25 (3,11, 5,48)	485,76 (358,22, 631,84)	4,17 (3,08, 5,43)	-0,68 (-1,19, -0,16)
Kenya	344,24 (295,34, 387,82)	2,1 (1,8, 2,37)	944,99 (755,71, 1142,73)	5,14 (4,11, 6,21)	1,54 (0,69, 2,39)
Kiribati	459,81 (348,54, 578,6)	1,98 (1,5, 2,49)	1346,41 (980,89, 1777,24)	2,68 (1,95, 3,54)	1,04 (0,93, 1,14)
Kuwait	11,19 (7,86, 14,82)	15,11 (10,62, 20,01)	26,52 (17,57, 36,86)	22,36 (14,81, 31,07)	0,98 (0,64, 1,33)
Kyrgyzstan	30,82 (23,27, 38,22)	1,75 (1,32, 2,17)	84,78 (60,77, 110,51)	1,92 (1,37, 2,5)	-0,54 (-1,54, 0,48)
Laos	71,25 (60,48, 81,66)	1,6 (1,36, 1,83)	105,5 (84,84, 127,08)	1,61 (1,3, 1,94)	-1,38 (-1,9, -0,85)
Latvia	185,92 (129,92, 257,33)	4,48 (3,13, 6,2)	297,93 (206,75, 410,54)	4,16 (2,89, 5,74)	-0,54 (-0,69, -0,39)
Lebanon	67,34 (56,87, 78,65)	2,53 (2,14, 2,96)	122,11 (92,63, 155,84)	6,38 (4,84, 8,14)	3,36 (2,91, 3,81)
Lesotho	75,93 (54,14, 100,04)	2,32 (1,65, 3,05)	165,6 (105,52, 229,57)	3,2 (2,04, 4,43)	1,42 (1,25, 1,6)
Liberia	108,47 (79,04, 139,06)	6 (4,37, 7,69)	300,62 (201,88, 419,56)	14,37 (9,65, 20,06)	3,84 (3,43, 4,24)
Libya	71,69 (52,85, 92,01)	3,65 (2,69, 4,68)	153,55 (107,38, 208,74)	3,21 (2,24, 4,36)	-0,78 (-0,96, -0,6)
Lithuania	52,22 (34,88, 71,9)	1,23 (0,82, 1,7)	180,58 (119,85, 260,7)	2,68 (1,78, 3,87)	2,93 (2,53, 3,33)
Luxembourg	62,72 (52,01, 74,33)	1,71 (1,42, 2,02)	82,47 (63,28, 105,77)	2,95 (2,26, 3,79)	1,82 (1,57, 2,07)
Madagascar	21,78 (18,3, 25,47)	5,71 (4,8, 6,68)	25,65 (20,33, 31,05)	4,15 (3,29, 5,02)	-1,15 (-1,27, -1,02)
Malawi	330,41 (240,8, 430,29)	2,76 (2,01, 3,6)	631,42 (437,56, 866,2)	2,37 (1,64, 3,25)	-0,7 (-0,84, -0,56)
Malaysia	239,07 (170,98, 304,05)	2,5 (1,79, 3,18)	416,69 (298,68, 552,87)	2,26 (1,62, 3)	-0,83 (-1,1, -0,56)
Maldives	526,66 (406,85, 644,72)	2,98 (2,3, 3,65)	660,54 (439,36, 933,19)	2,11 (1,4, 2,98)	-2,11 (-2,61, -1,61)
Mali	5,35 (3,85, 7,28)	2,41 (1,73, 3,28)	10,51 (7,56, 13,8)	2,11 (1,52, 2,77)	-0,75 (-1,03, -0,48)
Malta	292,31 (220,89, 371,45)	3,37 (2,55, 4,28)	679,59 (489,82, 883,51)	3,1 (2,23, 4,03)	-0,48 (-0,61, -0,35)
Marshall Islands	36,74 (30,81, 42,93)	9,91 (8,31, 11,58)	45,98 (37,21, 55,31)	10,47 (8,47, 12,59)	0,33 (0,07, 0,59)
Mauritania	2,69 (1,92, 3,56)	5,88 (4,2, 7,79)	7,44 (4,96, 10,68)	13,1 (8,73, 18,78)	2,89 (2,78, 3)
Mauritius	98,64 (77,26, 123,91)	4,77 (3,74, 6)	191,53 (139,28, 254,27)	4,77 (3,47, 6,33)	0 (-0,05, 0,04)
Mexico	84,57 (67,62, 101,57)	7,69 (6,15, 9,23)	439,26 (317,6, 564,24)	34,41 (24,88, 44,2)	7,38 (6,4, 8,37)
Micronesia	6255,2 (4992,11, 7435,87)	7,32 (5,84, 8,7)	17830,33 (13589,88, 22187,17)	14,27 (10,88, 17,76)	2,22 (1,98, 2,45)

Moldova	8,97 (6,18, 12,43)	8,6 (5,92, 11,92)	22,99 (14,5, 33,22)	22,51 (14,2, 32,53)	3,36 (3,17, 3,55)
Monaco	0,94 (0,7, 1,18)	3,07 (2,31, 3,89)	1,37 (1,06, 1,7)	3,66 (2,81, 4,54)	0,66 (0,46, 0,86)
Mongolia	20,4 (15,96, 26,3)	0,95 (0,74, 1,22)	52,05 (38,71, 69,56)	1,54 (1,14, 2,05)	1,53 (1,44, 1,62)
Montenegro	21,32 (16,51, 26,11)	3,41 (2,64, 4,17)	41,32 (31,58, 51,53)	6,66 (5,09, 8,31)	2,51 (2,27, 2,75)
Morocco	341,13 (233,4, 483,57)	1,35 (0,92, 1,91)	1198,24 (803,16, 1644,71)	3,33 (2,23, 4,57)	3,48 (3,19, 3,77)
Mozambique	454,93 (336,36, 582,04)	3,48 (2,57, 4,45)	985,57 (703,7, 1340,74)	3,34 (2,38, 4,54)	0,19 (-0,02, 0,4)
Myanmar	2898,29 (2098,95, 3841,79)	7,05 (5,11, 9,35)	4729,39 (3482,28, 6007,7)	8,65 (6,37, 10,99)	0,56 (0,45, 0,67)
Namibia	113,29 (85,07, 141,41)	8,04 (6,03, 10,03)	220,4 (160,17, 292,75)	9,17 (6,67, 12,18)	0,03 (-0,33, 0,39)
Nauru	0,78 (0,53, 1,09)	7,56 (5,15, 10,62)	1,23 (0,8, 1,88)	11,65 (7,6, 17,82)	1,24 (0,9, 1,59)
Nepal	237,24 (175,57, 311,56)	1,21 (0,9, 1,59)	877,48 (632,4, 1131,64)	2,88 (2,08, 3,72)	3,33 (2,91, 3,76)
Netherlands	1184,69 (979,31, 1374,25)	7,94 (6,56, 9,21)	1138,72 (926,91, 1347,73)	6,64 (5,4, 7,86)	-1,12 (-1,47, -0,76)
New Zealand	143,57 (120,5, 166,07)	4,2 (3,53, 4,86)	236,57 (198,19, 276,25)	5,26 (4,41, 6,14)	-0,12 (-0,57, 0,33)
Nicaragua	97,51 (75,86, 120,51)	2,51 (1,95, 3,1)	446,69 (332,72, 566,46)	6,86 (5,11, 8,7)	3,08 (2,73, 3,44)
Niger	173,31 (128,66, 227,13)	2,16 (1,6, 2,83)	521,69 (374,11, 686,6)	2,24 (1,61, 2,95)	0,01 (-0,12, 0,14)
Nigeria	2675,22 (1981,16, 3432,71)	2,97 (2,2, 3,81)	5115,81 (3683,52, 6805,24)	2,38 (1,71, 3,17)	-0,85 (-0,89, -0,81)
Niue	0,4 (0,28, 0,55)	17,4 (12,18, 23,74)	0,52 (0,35, 0,71)	30,91 (21, 42,59)	1,99 (1,57, 2,42)
North Korea	89,51 (69,05, 111,14)	4,44 (3,43, 5,51)	269,72 (191,51, 352,35)	12,53 (8,9, 16,37)	3,91 (3,47, 4,36)
North Macedonia	1,57 (1,12, 2,1)	3,46 (2,47, 4,63)	5,47 (3,82, 7,3)	12,86 (9, 17,17)	5,36 (4,83, 5,9)
Northern Mariana Islands	189,66 (155,42, 224,64)	4,47 (3,66, 5,29)	235,89 (190,43, 284,37)	4,41 (3,56, 5,32)	-0,22 (-0,61, 0,18)
Norway	50,79 (33,18, 70,43)	2,61 (1,71, 3,62)	131,52 (92,18, 171,46)	2,87 (2,01, 3,74)	0,67 (0,22, 1,13)
Oman	3858,22 (2460,98, 4959,13)	3,42 (2,18, 4,39)	14751,63 (11157,24, 18953,76)	6,58 (4,98, 8,46)	2,28 (2,14, 2,42)
Pakistan	1,43 (0,97, 1,99)	9,28 (6,29, 12,9)	4,59 (3,05, 6,42)	25,51 (16,95, 35,65)	3,59 (3,53, 3,65)
Palau	108,07 (78,5, 142,71)	5,22 (3,79, 6,89)	306,8 (232,74, 388,41)	6,19 (4,7, 7,84)	0,56 (-0,04, 1,17)
Palestine	67,01 (51,83, 81,75)	2,81 (2,17, 3,42)	299,3 (214,19, 399,63)	7,19 (5,15, 9,61)	3,01 (2,71, 3,31)
Panama	287,28 (195,36, 393,06)	7,03 (4,78, 9,62)	959,56 (654,79, 1308,97)	9,73 (6,64, 13,27)	1,01 (0,93, 1,1)
Papua New Guinea	122,9 (96,2, 150,28)	3,04 (2,38, 3,71)	590,52 (424,82, 780,43)	8,52 (6,13, 11,26)	3,63 (3,18, 4,08)
Paraguay	330,61 (245,11, 428,87)	1,52 (1,13, 1,97)	860,53 (557,86, 1238,55)	2,53 (1,64, 3,64)	1,93 (1,61, 2,25)
Peru	1895,76 (1456,59, 2333,09)	3 (2,3, 3,69)	6166,9 (4441,88, 8146,09)	5,5 (3,96, 7,26)	2,07 (1,88, 2,25)
Philippines	1784,73 (1505,04, 2049,07)	4,68 (3,94, 5,37)	2529,77 (1975,68, 3105,87)	6,58 (5,14, 8,08)	1,67 (1,17, 2,16)
Poland	775,05 (611,66, 928,1)	7,65 (6,03, 9,16)	1328,63 (1051,36, 1589,32)	12,47 (9,87, 14,92)	1,22 (0,85, 1,59)
Portugal	359,32 (271,6, 442,73)	9,94 (7,52, 12,25)	656,27 (447,88, 880,28)	18,64 (12,72, 25)	1,59 (1,34, 1,83)
Puerto Rico	12,65 (9,09, 16,46)	2,84 (2,04, 3,7)	71,04 (46,04, 99,74)	2,48 (1,61, 3,48)	-1,29 (-2,19, -0,38)
Qatar	1188,67 (915,45, 1500,99)	2,68 (2,06, 3,39)	3578,62 (2822,64, 4382,22)	6,7 (5,29, 8,21)	2,43 (1,47, 3,39)
Romania	91,91 (74,22, 108,88)	2,07 (1,67, 2,45)	83,76 (64,94, 104,82)	2,27 (1,76, 2,84)	-0,68 (-1,1, -0,25)
Russia	549,83 (443,59, 649,7)	2,35 (1,9, 2,78)	808,53 (626,98, 1021,97)	4,2 (3,26, 5,31)	2,25 (1,9, 2,6)

Rwanda	3128,29 (2642,88, 3626,86)	2,07 (1,75, 2,4)	6299,55 (5035,78, 7631,35)	4,29 (3,43, 5,2)	1,91 (1,1, 2,73)
Saint Kitts and Nevis	183,68 (114,92, 260,1)	2,56 (1,6, 3,63)	259,21 (161,18, 381,14)	2,04 (1,27, 3)	-1,7 (-2,26, -1,13)
Saint Lucia	7,21 (5,77, 8,66)	17,44 (13,95, 20,94)	8,79 (6,8, 10,99)	14,76 (11,43, 18,46)	-0,81 (-1,12, -0,51)
Saint Vincent and the Grenadines	16,07 (12,09, 19,65)	11,7 (8,8, 14,3)	30,33 (22,62, 38,88)	17,37 (12,96, 22,27)	0,61 (0,26, 0,96)
Samoa	13,96 (10,68, 17,22)	12,69 (9,71, 15,65)	20,15 (14,33, 26,2)	17,81 (12,66, 23,16)	0,62 (0,31, 0,93)
San Marino	12,52 (8,65, 17,12)	7,65 (5,29, 10,46)	26,04 (18,55, 35,05)	12,32 (8,78, 16,58)	1,64 (1,28, 2)
Sao Tome and Principe	0,84 (0,65, 1,05)	3,57 (2,76, 4,47)	1,62 (1,07, 2,23)	4,89 (3,23, 6,73)	1,25 (1,12, 1,37)
Saudi Arabia	1,29 (0,93, 1,67)	1,07 (0,77, 1,37)	2,23 (1,55, 2,91)	1,09 (0,76, 1,42)	-0,18 (-0,31, -0,05)
Senegal	245,21 (154,06, 347,06)	1,53 (0,96, 2,16)	642,02 (445,68, 876,45)	1,8 (1,25, 2,45)	-0,27 (-0,68, 0,14)
Serbia	228,03 (172,67, 290,65)	2,99 (2,27, 3,81)	637,15 (454,3, 830,38)	4,21 (3, 5,49)	1,26 (1,09, 1,43)
Seychelles	540,64 (398,13, 683,89)	5,75 (4,24, 7,28)	896,81 (632,42, 1173,57)	10,25 (7,23, 13,42)	2,14 (1,88, 2,39)
Sierra Leone	1,69 (1,27, 2,16)	2,32 (1,74, 2,95)	4,7 (3,48, 5,97)	4,6 (3,41, 5,85)	2,3 (2,16, 2,44)
Singapore	112,97 (84,8, 144,31)	3,09 (2,32, 3,95)	258,42 (183,96, 350,26)	3,12 (2,22, 4,23)	-0,26 (-0,42, -0,11)
Slovakia	89,3 (67,96, 108,18)	2,93 (2,23, 3,55)	44,95 (34,73, 55,11)	0,79 (0,61, 0,97)	-4,98 (-6,03, -3,92)
Slovenia	245,71 (200,11, 293,01)	4,65 (3,79, 5,55)	266,36 (196,44, 343,09)	4,9 (3,61, 6,31)	0,34 (0,14, 0,55)
Solomon Islands	86,87 (64,57, 112,42)	4,41 (3,28, 5,7)	120,35 (88,52, 159,02)	5,8 (4,27, 7,67)	-1,24 (-2,15, -0,33)
Somalia	22,9 (15,22, 34,22)	6,73 (4,47, 10,05)	82,26 (56,86, 111,66)	12,55 (8,67, 17,03)	2,08 (1,9, 2,27)
South Africa	266,88 (183,83, 363,29)	3,73 (2,57, 5,08)	654,14 (459,09, 897,88)	3,22 (2,26, 4,41)	-0,41 (-0,47, -0,35)
South Korea	2142,82 (1663,91, 2630,95)	5,82 (4,52, 7,14)	7464,15 (6035,84, 8903,35)	13,43 (10,86, 16,02)	3,34 (2,92, 3,77)
South Sudan	138,84 (93,93, 197,1)	2,37 (1,6, 3,36)	201,36 (128,71, 289,56)	2,17 (1,39, 3,12)	-0,59 (-0,91, -0,26)
Spain	3314,65 (2694,1, 3880,75)	8,55 (6,95, 10,01)	3452,73 (2729,62, 4188,85)	7,5 (5,93, 9,1)	-0,97 (-1,17, -0,77)
Sri Lanka	444,94 (333,24, 557,33)	2,58 (1,94, 3,24)	2388,71 (1588,27, 3264,25)	10,93 (7,27, 14,94)	5,88 (5,3, 6,46)
Sudan	247,67 (168,76, 363,23)	1,23 (0,84, 1,8)	623,84 (388,78, 926,04)	1,53 (0,95, 2,27)	0,94 (0,73, 1,14)
Suriname	19,66 (14,68, 24,36)	5,08 (3,8, 6,3)	55,39 (39,13, 72)	9,62 (6,79, 12,5)	2,1 (1,7, 2,5)
Sweden	510,23 (422,36, 595,31)	5,94 (4,92, 6,93)	737,79 (595,2, 872,68)	7,22 (5,82, 8,54)	0,63 (0,36, 0,9)
Switzerland	542,34 (450,41, 627,77)	7,9 (6,56, 9,14)	497,08 (405,06, 588,52)	5,66 (4,62, 6,71)	-1,88 (-2,2, -1,57)
Syria	172,76 (117,99, 234,1)	1,34 (0,91, 1,82)	337,96 (224,56, 465)	2,33 (1,55, 3,21)	1,15 (0,24, 2,06)
Taiwan (Province of China)	1085,95 (834,6, 1316,58)	5,32 (4,09, 6,45)	2746,89 (1986,53, 3643,61)	11,63 (8,41, 15,43)	1,71 (1,13, 2,29)
Tajikistan	109,12 (88,05, 130,85)	2,03 (1,64, 2,43)	478,56 (363,73, 618,62)	5,04 (3,83, 6,52)	3,28 (2,8, 3,76)

Tanzania	1578,79 (1113,13, 2057,5)	2,78 (1,96, 3,62)	3283,52 (2120,22, 4525,99)	4,68 (3,02, 6,46)	1,03 (0,5, 1,56)
Thailand	12,36 (8,68, 17,15)	1,58 (1,11, 2,19)	42,54 (30,35, 57,56)	3,19 (2,27, 4,31)	2,75 (2,59, 2,91)
Timor-Leste	69,32 (53,13, 88,42)	1,89 (1,45, 2,41)	241,32 (172,47, 327,52)	3,05 (2,18, 4,13)	1,57 (1,44, 1,7)
Togo	0,22 (0,15, 0,29)	12,96 (9,13, 17,46)	0,25 (0,17, 0,34)	17,65 (11,83, 24,18)	1,14 (0,79, 1,49)
Tokelau	9,57 (7,04, 12,49)	9,88 (7,28, 12,9)	16,58 (11,45, 22,53)	16,2 (11,19, 22,01)	1,76 (1,57, 1,94)
Tonga	237,71 (183,69, 285,88)	19,76 (15,27, 23,76)	431,55 (297,78, 582,75)	31,1 (21,46, 42)	1,02 (0,77, 1,28)
Trinidad and Tobago	95,37 (64,95, 133,73)	1,13 (0,77, 1,58)	328,37 (210,62, 469,49)	2,84 (1,82, 4,06)	3,36 (3,24, 3,48)
Tunisia	2441,75 (1731,55, 3178,45)	4,09 (2,9, 5,32)	3628,34 (2566,57, 4844,54)	4,46 (3,15, 5,95)	0,66 (0,3, 1,02)
Turkey	70,79 (59,15, 81,69)	1,91 (1,6, 2,2)	268,05 (204,84, 350,73)	5,27 (4,03, 6,9)	2,96 (2,6, 3,33)
Turkmenistan	1,3 (0,92, 1,74)	13,92 (9,84, 18,65)	2,36 (1,59, 3,42)	19,98 (13,48, 28,99)	1,07 (0,84, 1,3)
Tuvalu	284,72 (180,59, 415,06)	1,64 (1,04, 2,4)	674,59 (435,24, 963,6)	1,64 (1,06, 2,34)	-0,37 (-0,51, -0,23)
Uganda	859,76 (710,67, 995,76)	1,63 (1,35, 1,89)	736,17 (554,39, 920,28)	1,67 (1,26, 2,09)	-1,26 (-1,7, -0,81)
UK	45,65 (32,81, 60,44)	2,44 (1,75, 3,23)	257,68 (161,41, 379,32)	2,79 (1,75, 4,1)	-1,04 (-1,78, -0,29)
Ukraine	2963,28 (2530,03, 3377,15)	5,16 (4,4, 5,88)	2363,06 (1978,51, 2768,39)	3,52 (2,94, 4,12)	-1,61 (-1,83, -1,39)
United Arab Emirates	659,46 (478,56, 846,72)	2,55 (1,85, 3,27)	1322,31 (913,16, 1794,28)	2,33 (1,61, 3,16)	-0,47 (-0,56, -0,37)
Uruguay	16574,2 (14104,42, 19041,39)	6,54 (5,56, 7,51)	28113,25 (23561,17, 32600,02)	8,57 (7,18, 9,94)	0,25 (-0,18, 0,69)
USA	6,34 (4,57, 8,26)	5,98 (4,31, 7,79)	15,12 (10,81, 19,67)	14,54 (10,39, 18,92)	3,3 (3,07, 3,53)
Uzbekistan	261,96 (223, 299,19)	8,34 (7,1, 9,53)	336,51 (279,92, 390,91)	9,79 (8,15, 11,38)	0,63 (0,46, 0,81)
Vanuatu	352,79 (293,85, 413,52)	1,68 (1,4, 1,97)	2455,21 (1900,62, 3088,82)	7,29 (5,64, 9,17)	4,49 (4,01, 4,97)
Venezuela	5,45 (3,44, 8,54)	3,6 (2,27, 5,64)	25,79 (16,81, 36,68)	8,76 (5,71, 12,45)	3,33 (3,15, 3,51)
Vietnam	674,41 (523,54, 834,71)	3,58 (2,78, 4,43)	2545,47 (1757,1, 3425,23)	9,07 (6,26, 12,2)	2,92 (2,58, 3,27)
Virgin Islands US	2856,47 (2047,92, 3712,64)	4,2 (3,01, 5,46)	6336,05 (4507,98, 8404,14)	6,57 (4,68, 8,72)	1,74 (1,47, 2)
Yemen	135,04 (88,57, 206,73)	0,98 (0,65, 1,51)	461,41 (309,69, 667,83)	1,46 (0,98, 2,12)	1,53 (1,32, 1,73)
Zambia	249,07 (188,53, 317,04)	3,14 (2,37, 3,99)	570,14 (420,25, 752,3)	3,13 (2,3, 4,12)	-0,52 (-0,79, -0,26)
Zimbabwe	281,44 (222,21, 346,94)	2,72 (2,15, 3,36)	786,48 (559,75, 1047,85)	5,24 (3,73, 6,98)	2,68 (2,29, 3,07)

No., number; ASMR, age-standardized mortality rate; UI, uncertainty interval; EAPC, estimated annual percentage change; CI, confidence interval.

Table S4. The number of cases and ASR of DALYs attributable to diet-related T2DM in 204 countries and territories in 1990 and 2019 and the change of DALYs between 1990 and 2019 (Source: Institute for Health Metrics and Evaluation (IHME). **GBD Results**. Seattle, WA: IHME, University of Washington, 2020. Available from https://vizhub.healthdata.org/gbd-results/)

Country	1990		2019		1990-2019
	DALYs No. (95% *UI*)	ASDR per 100,000 No. (95% *UI*)	DALYs No. (95% *UI*)	ASDR per 100,000 No. (95% *UI*)	EAPC in ASDR No. (95% *CI*)
Afghanistan	21575,11 (14617,37, 30956,11)	188,94 (128,01, 271,1)	63359,55 (43164,6, 88202,98)	165,53 (112,77, 230,43)	0,85 (-1,84, 3,63)
Albania	2135,7 (1535,35, 2923,12)	64,52 (46,39, 88,31)	5502,67 (3774,96, 7603,94)	202,28 (138,77, 279,52)	2,82 (-0,42, 6,17)
Algeria	20374,13 (13970,99, 27730,58)	80,59 (55,26, 109,68)	70729,92 (45591,18, 98396,07)	169,02 (108,95, 235,13)	4,98 (1,64, 8,42)
American Samoa	143,27 (101,54, 189,21)	295,82 (209,65, 390,69)	391,42 (276,22, 519,21)	705,2 (497,66, 935,42)	-1,27 (-4,61, 2,18)
Andorra	59,8 (43,25, 80,02)	110,55 (79,95, 147,93)	201,08 (141,06, 276,14)	242,08 (169,82, 332,44)	0,3 (-2,52, 3,21)
Angola	14336,51 (10344,21, 18883,99)	138,94 (100,25, 183,01)	35932,93 (25468,79, 48494,12)	119,23 (84,51, 160,9)	-0,11 (-2,78, 2,62)
Antigua and Barbuda	233,04 (178,11, 289,2)	383,77 (293,32, 476,26)	487,49 (358,14, 629,36)	550,9 (404,73, 711,22)	1,28 (-1,45, 4,09)
Argentina	79678,51 (64109,19, 95428,92)	240,57 (193,56, 288,12)	151864,41 (118240,11, 190976,01)	336,61 (262,08, 423,31)	1,06 (-1,61, 3,8)
Armenia	5808,89 (4458,64, 7307,95)	170,12 (130,58, 214,02)	12898,77 (9392,47, 16428,91)	427,16 (311,04, 544,06)	3,58 (-0,02, 7,31)
Australia	28173,96 (22422,36, 34888,49)	167,1 (132,99, 206,93)	67448,47 (50157, 87786,42)	274,54 (204,15, 357,32)	0,62 (-2,74, 4,09)
Austria	15377,82 (12057,33, 18940,12)	197,95 (155,21, 243,81)	28194,55 (21025,63, 36817,25)	316,22 (235,81, 412,93)	1,88 (-0,57, 4,38)
Azerbaijan	8284,69 (6210,1, 10509,4)	113,01 (84,71, 143,36)	30723,7 (22284,86, 39950,47)	298,91 (216,81, 388,67)	-0,19 (-3,12, 2,83)
Bahamas	682,57 (521,16, 859,26)	266,15 (203,21, 335,04)	1675,13 (1241,85, 2213,94)	444,4 (329,46, 587,35)	2,35 (-1,04, 5,86)
Bahrain	755,14 (527,41, 996,36)	148,64 (103,81, 196,12)	6315,18 (4230,65, 8576,02)	437,74 (293,25, 594,45)	0,6 (-2,41, 3,72)
Bangladesh	116496,5 (90608,54, 143633,33)	106,82 (83,09, 131,71)	328006,11 (246601,12, 420547,73)	205,96 (154,84, 264,06)	3,24 (0,02, 6,56)
Barbados	1502,91 (1186,02, 1847,94)	591,94 (467,13, 727,83)	2243,81 (1641,89, 2886)	753,54 (551,39, 969,2)	2,6 (-0,87, 6,19)

Belarus	15828,03 (11734,9, 20593,67)	151,17 (112,08, 196,68)	15974,57 (10862,41, 22124,48)	168,14 (114,33, 232,87)	0,61 (-2,29, 3,59)
Belgium	22223,09 (16760,12, 28635,53)	222,69 (167,94, 286,94)	36613,57 (25793,46, 49606,18)	320,63 (225,88, 434,41)	0,36 (-2,76, 3,59)
Belize	193,76 (134,49, 254,6)	104,24 (72,35, 136,96)	878,39 (602,28, 1184,55)	214,19 (146,86, 288,85)	0,26 (-2,82, 3,44)
Benin	3886,12 (2907,29, 4848,13)	80,08 (59,91, 99,91)	12425,07 (8897,09, 16331,83)	98,1 (70,25, 128,94)	-0,26 (-3,35, 2,93)
Bermuda	147,75 (111,6, 187,47)	248,51 (187,71, 315,32)	283,87 (210,81, 367,33)	443,34 (329,23, 573,69)	2,22 (-0,89, 5,42)
Bhutan	479,56 (346,11, 616,98)	78,34 (56,54, 100,79)	1524,96 (1097,65, 2004,83)	202,18 (145,53, 265,8)	0,16 (-2,36, 2,74)
Bolivia	8636,66 (6125,43, 11488,12)	134,51 (95,4, 178,91)	27953,09 (20186,88, 36677,91)	232,71 (168,06, 305,35)	0,86 (-2,83, 4,7)
Bosnia and Herzegovina	6897,61 (4934,07, 8966,6)	152 (108,73, 197,59)	20091,3 (14254,66, 26515,96)	608,83 (431,96, 803,52)	-0,12 (-3,15, 3,02)
Botswana	2558,81 (1880,02, 3342,15)	196,58 (144,43, 256,76)	9443,92 (6595,66, 12589,48)	403,81 (282,02, 538,31)	-0,54 (-3,81, 2,85)
Brazil	262094,79 (203992,76, 330653,2)	176,1 (137,06, 222,16)	695189,88 (540328,12, 862618,26)	320,86 (249,38, 398,13)	3,16 (-0,92, 7,4)
Brunei	868,19 (661,33, 1084,2)	335,8 (255,79, 419,36)	2204,25 (1664,4, 2823,21)	504,27 (380,77, 645,87)	2,1 (-1,15, 5,45)
Bulgaria	28268,74 (21525,02, 35653,8)	325,63 (247,95, 410,7)	38066,97 (28367,03, 48885,19)	548,94 (409,06, 704,94)	2,13 (-1,09, 5,46)
Burkina Faso	11704,11 (8963,08, 14717,43)	122,46 (93,78, 153,99)	28453,98 (21379,66, 36009,27)	125,39 (94,22, 158,69)	2,17 (-0,65, 5,08)
Burundi	4887,81 (3254,52, 7180,67)	87,74 (58,42, 128,9)	8151,01 (5336,45, 11148,61)	68,3 (44,71, 93,42)	-0,29 (-2,79, 2,27)
Cambodia	259,89 (191,02, 340,06)	73,92 (54,33, 96,71)	1146,44 (841,3, 1488,49)	203,43 (149,28, 264,12)	1,03 (-1,57, 3,7)
Cameroon	12017,88 (8747,92, 15727,91)	115,83 (84,31, 151,59)	34939,74 (25673,79, 46333)	210,44 (154,63, 279,06)	1,02 (-2,45, 4,61)
Canada	8532,37 (6024,44, 11435,35)	82,11 (57,98, 110,05)	32592,17 (22322,98, 44889,48)	111,99 (76,71, 154,25)	-0,31 (-2,77, 2,21)
Cape Verde	35487,88 (28271,69, 43456,2)	130,2 (103,72, 159,43)	89149,76 (66729,86, 116910,25)	244,11 (182,72, 320,13)	-0,72 (-4,15, 2,83)
Central African Republic	4711,72 (3501,28, 6166,45)	171,71 (127,6, 224,72)	10563,41 (7593,56, 14049,33)	199,31 (143,28, 265,09)	1,67 (-1,59, 5,03)
Chad	6709,29 (5190,66, 8396,78)	111,41 (86,19, 139,43)	18861,68 (14089,82, 24110,68)	115,02 (85,92, 147,03)	-0,82 (-3,89, 2,36)
Chile	21442,17 (16756,47, 27237,76)	161,46 (126,18, 205,1)	68079,95 (50339,96, 88758,87)	374,1 (276,62, 487,73)	3,97 (1,45, 6,56)

China	1048787,03 (769115,04, 1372981,87)	88,6 (64,98, 115,99)	2522819,68 (1817116,69, 3372078,52)	177,37 (127,75, 237,08)	-0,53 (-4,16, 3,25)
Colombia	40741,48 (28262,13, 54143,47)	125,18 (86,83, 166,35)	113369,18 (77114,26, 158733,58)	237,29 (161,41, 332,24)	1,13 (-1,84, 4,19)
Comoros	385,91 (227,59, 541,21)	82,86 (48,86, 116,2)	924,37 (635,48, 1262,07)	129,4 (88,96, 176,67)	1,01 (-2,68, 4,85)
Congo	4336,25 (3173,36, 5557,19)	177,37 (129,8, 227,31)	10475,2 (7463,3, 13819,77)	198,93 (141,73, 262,44)	0,2 (-2,99, 3,5)
Cook Islands	86,11 (61,41, 113,78)	452,71 (322,84, 598,17)	167,32 (121,1, 220,84)	930,25 (673,28, 1227,81)	-0,17 (-3,03, 2,79)
Costa Rica	3593,28 (2447,15, 4838,16)	118,2 (80,5, 159,15)	11481,66 (7697,99, 15959,41)	243,42 (163,21, 338,36)	0,91 (-2,37, 4,31)
Cote d'Ivoire	8254,22 (5971,89, 10880,8)	67,51 (48,84, 88,99)	27971,85 (19695,03, 37024,94)	106,88 (75,25, 141,47)	0 (-3,71, 3,86)
Croatia	9183,01 (6295,08, 12344,26)	187,38 (128,45, 251,89)	15200,9 (10281,69, 20825,13)	357,84 (242,04, 490,25)	-0,02 (-3,18, 3,24)
Cuba	24799,71 (17445,51, 32945,36)	228,94 (161,05, 304,14)	34216,6 (22352,63, 48755,24)	301,24 (196,79, 429,24)	3,79 (0,3, 7,39)
Cyprus	3180,31 (2475,98, 3979,17)	408,78 (318,25, 511,46)	4850,31 (3606,25, 6247,46)	369,27 (274,56, 475,64)	-1,53 (-3,75, 0,74)
Czech	31655,12 (23318,35, 41612,13)	307,41 (226,45, 404,1)	70063,13 (49644,82, 94377,08)	658,27 (466,43, 886,71)	4,14 (1, 7,38)
Denmark	19748,51 (13532,62, 26685,91)	93,8 (64,27, 126,74)	45367,75 (31607,75, 61146,37)	172,94 (120,49, 233,09)	-0,04 (-3,01, 3,02)
Djibouti	34864,22 (24320,87, 46370,57)	90,34 (63,02, 120,16)	100489,21 (70529,05, 132376,32)	114,62 (80,45, 150,99)	1,64 (-1,62, 5,01)
Dominica	9046,37 (7370,09, 10937,8)	175,85 (143,27, 212,62)	16346,03 (12743,16, 20739,33)	281,7 (219,61, 357,41)	0,92 (-2,51, 4,47)
Dominican Republic	434,22 (313,95, 572,81)	89,34 (64,6, 117,86)	2158,27 (1536,06, 2865,93)	179,44 (127,71, 238,27)	-0,23 (-3,47, 3,12)
DR Congo	268,36 (195,87, 337,53)	362,63 (264,67, 456,09)	399,28 (283,07, 521,58)	581,34 (412,14, 759,42)	-0,42 (-3,98, 3,27)
Ecuador	5290,7 (3670,76, 6944,28)	73,45 (50,96, 96,41)	20175,88 (13737,02, 27599,29)	185,41 (126,24, 253,63)	3,91 (0,96, 6,95)
Egypt	9206,47 (6536,16, 11887,73)	91,82 (65,19, 118,57)	44952,35 (31504,98, 58800,57)	255,58 (179,12, 334,31)	5,29 (2,43, 8,24)
El Salvador	39388,07 (26847,69, 52467,14)	70,72 (48,2, 94,2)	146434,77 (95225,12, 204084,13)	147,81 (96,12, 206)	-0,19 (-3,28, 3)
Equatorial Guinea	4463,59 (3108,52, 5862,44)	84,74 (59,01, 111,29)	17300,24 (12033,58, 23472,59)	276,53 (192,35, 375,19)	1,93 (-1,14, 5,08)
Eritrea	680,7 (483,91, 905)	158,18 (112,45, 210,3)	1636,72 (1079,55, 2308,91)	115,28 (76,03, 162,62)	-0,97 (-3,82, 1,96)

Estonia	2857,44 (1967,58, 3984,71)	95,2 (65,56, 132,76)	8253,37 (5634,74, 11282,2)	122,98 (83,96, 168,11)	0,99 (-1,83, 3,89)
Eswatini	2260,67 (1613,97, 3047,24)	144,11 (102,89, 194,25)	3554,76 (2471,22, 4900,84)	270,87 (188,3, 373,44)	3,55 (0,35, 6,86)
Ethiopia	1608,21 (1187,76, 2066,82)	199,36 (147,24, 256,21)	4713,88 (3260,16, 6473,5)	412,73 (285,45, 566,8)	1,36 (-1,36, 4,15)
Fiji	90833,62 (67845,84, 118676,7)	176,75 (132,02, 230,93)	99079,89 (75431,54, 122171,45)	92,09 (70,11, 113,55)	2,1 (-1,36, 5,69)
Finland	4842,15 (3664,47, 6201,91)	637,68 (482,59, 816,76)	13925,03 (10250,26, 18082,21)	1528,13 (1124,86, 1984,33)	3,43 (0,38, 6,58)
France	9893,77 (7329,39, 12891,64)	197,47 (146,29, 257,31)	18781,72 (12657,55, 25998,5)	339,38 (228,72, 469,79)	0,18 (-3,13, 3,61)
Gabon	74548,6 (59721,53, 90559,56)	129,04 (103,38, 156,76)	145201,2 (112360,75, 183824,45)	219,32 (169,72, 277,66)	1,93 (-1,32, 5,29)
Gambia	2435,16 (1770,39, 3234,21)	245,59 (178,55, 326,18)	4639,96 (3361,78, 6172,47)	265,13 (192,1, 352,7)	1,92 (-0,63, 4,55)
Georgia	702,34 (509,15, 890,65)	70,8 (51,33, 89,79)	2747,76 (2042,3, 3511,08)	122,35 (90,94, 156,34)	2 (-1,28, 5,39)
Germany	9352,87 (6943,12, 12075,24)	169,79 (126,04, 219,21)	18763,46 (13788,91, 24350,82)	512 (376,26, 664,46)	3,96 (1,26, 6,72)
Ghana	262613,1 (204016,8, 336338,88)	328,51 (255,21, 420,73)	400132,61 (291647,84, 533485,47)	471,22 (343,46, 628,27)	1,06 (-2,25, 4,49)
Greece	11790,58 (8569,09, 15465,03)	78,52 (57,06, 102,99)	39077,58 (27165,87, 51832,83)	123,91 (86,14, 164,36)	2,58 (-0,11, 5,35)
Greenland	16950,58 (12460,55, 22152,19)	163,15 (119,93, 213,22)	32434,86 (22863,11, 43596,58)	313,77 (221,17, 421,75)	4,46 (1,22, 7,8)
Grenada	61,28 (48,9, 75,98)	110,25 (87,99, 136,7)	129,98 (96,13, 169,38)	231,32 (171,08, 301,45)	-1,06 (-4, 1,98)
Guam	283,62 (208,04, 361,86)	331,05 (242,83, 422,37)	529,84 (376,78, 703,53)	513,33 (365,04, 681,62)	1,09 (-1,87, 4,14)
Guatemala	217,97 (158,82, 287,87)	159,38 (116,13, 210,49)	516,95 (371,25, 689,28)	302,97 (217,58, 403,96)	0,1 (-2,18, 2,44)
Guinea	6074,72 (4184,52, 8000,95)	76,25 (52,53, 100,43)	51170,52 (35907,42, 67972,84)	287,86 (201,99, 382,37)	-0,78 (-4,28, 2,85)
Guinea-Bissau	5100,18 (3609,48, 6649,74)	82,44 (58,35, 107,49)	12674,96 (8948,16, 17072,5)	100,25 (70,77, 135,03)	0,7 (-1,83, 3,31)
Guyana	1310,81 (960,9, 1715,3)	130,13 (95,39, 170,28)	2757,79 (2018,89, 3681,49)	145,06 (106,19, 193,64)	0,15 (-2,94, 3,34)
Haiti	2275,02 (1684,22, 2872,95)	295,43 (218,71, 373,08)	4468,53 (3149,33, 5813,57)	579,8 (408,63, 754,32)	0,38 (-2,67, 3,53)
Honduras	14550,18 (9797,69, 20042,53)	228,93 (154,16, 315,35)	34030,03 (22410,86, 47884,28)	274,39 (180,7, 386,1)	3,8 (0,07, 7,67)

Hungary	3644,71 (2421,24, 5099,36)	77,41 (51,42, 108,3)	16501,07 (11039,67, 23491,24)	168,13 (112,48, 239,35)	4,02 (1,06, 7,07)
Iceland	27724,27 (20530,18, 36042,76)	266,77 (197,55, 346,81)	47475,22 (33880,32, 62569,25)	490,73 (350,21, 646,75)	3,15 (-0,11, 6,51)
India	288,42 (214,6, 377,83)	113,56 (84,5, 148,77)	779,75 (540,85, 1069,4)	226,1 (156,82, 310,08)	2,2 (-0,43, 4,91)
Indonesia	843594,71 (641616,83, 1070768,77)	98,6 (74,99, 125,15)	2941701,85 (2244263,26, 3721471,65)	211,53 (161,38, 267,6)	3,66 (0,99, 6,41)
Iran	242348,91 (186562,71, 297740,24)	130,73 (100,64, 160,61)	673088,88 (482992,69, 860729,78)	259,41 (186,15, 331,73)	1,16 (-2,06, 4,5)
Iraq	31002,68 (21821,55, 40668,1)	52,96 (37,28, 69,47)	148383,48 (106536,78, 195068,44)	176,02 (126,38, 231,4)	1,7 (-1,48, 4,98)
Ireland	25919,69 (18154,74, 34546,67)	147,3 (103,17, 196,33)	91036,14 (64438,62, 120950,21)	216,14 (152,99, 287,16)	2,13 (-0,72, 5,06)
Israel	4389,94 (3536,92, 5274,82)	121,9 (98,21, 146,47)	11122,72 (7914,26, 15054,37)	226,52 (161,17, 306,58)	-1,98 (-5,08, 1,22)
Italy	6332,1 (4739,67, 8051,49)	127,62 (95,52, 162,27)	22448,98 (16767,41, 29040,28)	241,14 (180,11, 311,94)	2,71 (-0,89, 6,43)
Jamaica	188423,86 (151577,48, 228691,98)	331,74 (266,87, 402,64)	282309,02 (213308,74, 359638,04)	468,07 (353,67, 596,28)	2,7 (-1,03, 6,58)
Japan	5666,53 (3958,48, 7318,8)	239,73 (167,47, 309,63)	13148,13 (9174,15, 17550,22)	467,78 (326,39, 624,4)	0,89 (-2,7, 4,6)
Jordan	173835,45 (126469,52, 227539,4)	138,11 (100,48, 180,78)	279792,07 (191660,83, 388757,31)	218,95 (149,98, 304,22)	1,87 (-1,86, 5,75)
Kazakhstan	5204,33 (3816,92, 6682,99)	137,93 (101,16, 177,12)	21502,08 (15356,91, 28119,33)	184,78 (131,97, 241,64)	1,85 (-1,02, 4,81)
Kenya	26256,5 (19295,4, 34043,2)	160,42 (117,89, 207,99)	61379,35 (45109,2, 79995,55)	333,73 (245,26, 434,95)	2,02 (-0,83, 4,95)
Kiribati	14984,26 (11327,55, 18768)	64,61 (48,84, 80,92)	50556,06 (37114,89, 65024,43)	100,65 (73,89, 129,46)	-0,87 (-3,76, 2,1)
Kuwait	396,25 (284,11, 519,68)	535 (383,6, 701,66)	991,15 (665,13, 1367,61)	835,56 (560,72, 1152,92)	2,35 (-1,01, 5,82)
Kyrgyzstan	1867,83 (1299,24, 2500,69)	106,16 (73,84, 142,13)	9320,86 (6228,77, 13458,47)	210,57 (140,71, 304,04)	2,13 (-0,99, 5,36)
Laos	4233,23 (3231,84, 5350,76)	94,87 (72,43, 119,91)	8258,51 (5984,86, 10816,92)	126,36 (91,58, 165,51)	1,32 (-1,77, 4,5)
Latvia	7003,47 (4973,88, 9490,04)	168,69 (119,81, 228,59)	13203,39 (8972,06, 17804,87)	184,45 (125,34, 248,73)	0,09 (-2,59, 2,85)
Lebanon	4560,2 (3357,95, 6052,45)	171,52 (126,3, 227,65)	6613,82 (4684,65, 8819,23)	345,32 (244,59, 460,46)	-3,2 (-6,4, 0,11)

Lesotho	3553,39 (2466,13, 4835,87)	108,5 (75,3, 147,66)	10812,39 (7257,39, 15237,93)	208,85 (140,18, 294,34)	2,55 (-0,11, 5,28)
Liberia	3173,64 (2313,26, 4075,43)	175,61 (128, 225,51)	8964,55 (6073,61, 12347,27)	428,6 (290,38, 590,33)	1,69 (-1,19, 4,66)
Libya	2452,79 (1772,74, 3136,85)	124,87 (90,25, 159,7)	6390,76 (4581,31, 8393,55)	133,42 (95,65, 175,23)	-0,15 (-3,32, 3,13)
Lithuania	3030,14 (2063,52, 4070,21)	71,52 (48,7, 96,07)	13846,7 (9028,9, 19526,56)	205,58 (134,05, 289,9)	1 (-2,11, 4,21)
Luxembourg	4912,14 (3529,42, 6576,29)	133,72 (96,08, 179,02)	6573,63 (4573,87, 9004,04)	235,26 (163,69, 322,24)	0,22 (-2,52, 3,05)
Madagascar	659,31 (526,93, 816,76)	172,93 (138,21, 214,23)	2237,98 (1573,05, 3083,91)	361,81 (254,31, 498,57)	0,89 (-2,47, 4,36)
Malawi	11314,23 (8300,82, 14584,63)	94,67 (69,45, 122,03)	26033,36 (18664,45, 34663,39)	97,54 (69,93, 129,87)	0,37 (-2,74, 3,58)
Malaysia	8147,67 (5860,59, 10385,37)	85,27 (61,33, 108,68)	15987,09 (11217,74, 21302,4)	86,69 (60,83, 115,51)	-1,43 (-3,99, 1,21)
Maldives	21999,56 (16361,9, 28363,98)	124,61 (92,68, 160,66)	47661,05 (31533,1, 67023,84)	152,26 (100,74, 214,12)	1,89 (-1,5, 5,39)
Mali	203,43 (143,67, 270,36)	91,67 (64,74, 121,83)	560,5 (389,11, 749,93)	112,46 (78,07, 150,46)	1,56 (-1,26, 4,46)
Malta	9823,06 (7379,06, 12333,62)	113,26 (85,08, 142,21)	24572,06 (17793,93, 31312,82)	112,11 (81,19, 142,87)	2,5 (-0,15, 5,22)
Marshall Islands	1193,68 (943,9, 1496,03)	322,02 (254,63, 403,58)	2172,51 (1590,8, 2864,9)	494,63 (362,19, 652,27)	0,29 (-3,32, 4,02)
Mauritania	112,83 (80,99, 147,24)	246,74 (177,12, 321,99)	369,25 (258,01, 493,84)	649,61 (453,9, 868,79)	-0,27 (-3,57, 3,13)
Mauritius	2870,18 (2220,61, 3547,51)	138,9 (107,46, 171,68)	5804,58 (4299,28, 7482,54)	144,6 (107,1, 186,4)	2 (-0,94, 5,04)
Mexico	3249,75 (2506,38, 3941,59)	295,41 (227,84, 358,3)	14033 (10226,73, 18003,55)	1099,19 (801,05, 1410,2)	-0,83 (-3,72, 2,15)
Micronesia	245300,1 (190169,13, 304561,88)	286,94 (222,45, 356,26)	702121,07 (534786,19, 897452,35)	561,97 (428,03, 718,31)	2,73 (-0,81, 6,4)
Moldova	305,04 (209,34, 412,97)	292,51 (200,74, 396,01)	830,22 (536,94, 1175,87)	813,02 (525,81, 1151,51)	2,55 (-0,37, 5,57)
Monaco	51,75 (37,07, 69,7)	170,01 (121,79, 228,96)	116,82 (80,33, 163,73)	310,93 (213,8, 435,79)	0,25 (-2,91, 3,5)
Mongolia	1177,86 (888,93, 1507,78)	54,69 (41,27, 70,01)	4013,69 (2928,7, 5320,41)	118,48 (86,45, 157,06)	-0,86 (-4,18, 2,58)
Montenegro	1220,43 (875,44, 1627,17)	195,04 (139,91, 260,04)	2624,93 (1865,84, 3537,79)	423,14 (300,78, 570,3)	1,36 (-1,56, 4,37)
Morocco	16952,8 (11706,4, 23051,24)	67,02 (46,28, 91,12)	64319,48 (42506,44, 88047,64)	178,9 (118,23, 244,9)	0,34 (-2,66, 3,44)
Mozambique	15138,84 (11351,37, 19071,77)	115,82 (86,84, 145,91)	36227,82 (25662,52, 47606,48)	122,69 (86,91, 161,22)	-1,47 (-4,33, 1,47)

Myanmar	99544,7 (72369,31, 131946,89)	242,18 (176,06, 321,01)	173114,9 (126432,82, 223206,36)	316,61 (231,24, 408,23)	1,99 (-0,99, 5,05)
Namibia	3312,61 (2542,76, 4132,91)	234,99 (180,38, 293,18)	6580,2 (4877,76, 8467,88)	273,82 (202,98, 352,37)	-1,99 (-5,24, 1,37)
Nauru	29,55 (20,2, 41,66)	288,13 (196,97, 406,24)	51,71 (34,83, 76,29)	490,09 (330,09, 723,03)	-0,62 (-3,32, 2,16)
Nepal	13795,31 (10038,54, 18099,51)	70,61 (51,38, 92,64)	48032,79 (33846,46, 63485,98)	157,92 (111,28, 208,72)	2,72 (0,04, 5,47)
Netherlands	35413,61 (28153,35, 43397,53)	237,3 (188,65, 290,8)	47663,27 (35349,43, 62492)	277,81 (206,04, 364,24)	0,99 (-2,8, 4,93)
New Zealand	5038,48 (4030,19, 6168,3)	147,44 (117,93, 180,5)	10703,92 (8246,24, 13751,22)	238,09 (183,43, 305,88)	2,33 (-0,96, 5,73)
Nicaragua	4375,17 (3247,31, 5625,73)	112,56 (83,54, 144,73)	18706,02 (13544,02, 24386,02)	287,33 (208,04, 374,57)	1,21 (-1,83, 4,34)
Niger	5413,49 (4093,49, 7002,75)	67,48 (51,02, 87,28)	18257,53 (13534,63, 23558,82)	78,37 (58,1, 101,13)	3,05 (-0,43, 6,66)
Nigeria	82076,56 (61170,22, 104795,63)	91,01 (67,83, 116,2)	168085,62 (122144,59, 220912,99)	78,24 (56,86, 102,83)	3,64 (0,15, 7,25)
Niue	11,79 (8,3, 15,73)	506,58 (356,9, 676,19)	17,23 (11,81, 23,38)	1030,6 (706,81, 1398,41)	0,57 (-2,54, 3,78)
North Korea	3997,62 (2910,49, 5216,3)	198,35 (144,41, 258,82)	11273,53 (8124,44, 14835,93)	523,69 (377,4, 689,17)	2,38 (-0,54, 5,39)
North Macedonia	78,48 (56,34, 103,03)	172,87 (124,11, 226,95)	248,59 (173,47, 333,13)	585 (408,22, 783,94)	2,78 (-0,52, 6,2)
Northern Mariana Islands	9097,78 (6866,56, 11814,32)	214,22 (161,68, 278,19)	14911,24 (10767,07, 19947,7)	278,77 (201,3, 372,93)	1,22 (-1,32, 3,83)
Norway	1890,74 (1258,02, 2510,59)	97,3 (64,74, 129,2)	6274,35 (4336,12, 8353,08)	136,88 (94,59, 182,22)	3,96 (0,81, 7,22)
Oman	155942,88 (114603,61, 198025,73)	138,2 (101,57, 175,5)	603331,6 (461821,21, 776479,08)	269,27 (206,11, 346,55)	-0,25 (-3,07, 2,66)
Pakistan	51,04 (35,09, 68,98)	331,32 (227,78, 447,82)	179,82 (121,2, 246,54)	998,59 (673,04, 1369,09)	3,5 (0,06, 7,06)
Palau	3147,16 (2297,33, 4121,7)	152,03 (110,98, 199,11)	10822,1 (8072,02, 13923,9)	218,34 (162,85, 280,92)	-0,77 (-3,71, 2,26)
Palestine	3199,61 (2341,65, 4204,86)	133,96 (98,04, 176,05)	13767,52 (9855,29, 18128,5)	330,91 (236,88, 435,73)	2,23 (-1,17, 5,75)
Panama	10902,07 (7563,09, 14682,26)	266,72 (185,03, 359,2)	39317,34 (26822,45, 52332,77)	398,49 (271,85, 530,4)	3,9 (0,59, 7,32)
Papua New Guinea	4514,94 (3455,49, 5651,21)	111,6 (85,42, 139,69)	19797,59 (14668,95, 25633,95)	285,66 (211,66, 369,87)	3 (0,23, 5,84)
Paraguay	13064,55 (9430,25, 16980,58)	60,12 (43,4, 78,14)	37469,76 (25229,85, 52188,94)	110,22 (74,22, 153,52)	2,48 (0,05, 4,98)
Peru	73047,11 (55337,09, 91442,61)	115,42 (87,44, 144,48)	236746,13 (175185,38, 301246,42)	211,11 (156,22, 268,63)	-0,5 (-3,82, 2,93)

Philippines	95392,23 (74480,73, 120105,91)	249,99 (195,19, 314,76)	161893,46 (117793,99, 210903,85)	421,22 (306,48, 548,74)	-1,4 (-4,7, 2,02)
Poland	26059,57 (19314,61, 33023,34)	257,07 (190,53, 325,77)	50896,82 (37470,33, 66135,32)	477,85 (351,79, 620,92)	4,11 (0,52, 7,83)
Portugal	12476 (9220,47, 16163,56)	345,28 (255,18, 447,33)	23096,23 (15976,89, 30827,98)	655,88 (453,7, 875,44)	0,11 (-2,29, 2,57)
Puerto Rico	555,66 (383,39, 746,63)	124,83 (86,13, 167,73)	6560,45 (4397,3, 9197,1)	229,02 (153,51, 321,07)	-0,54 (-3,08, 2,07)
Qatar	53811,76 (39834,19, 68592,34)	121,37 (89,84, 154,7)	155731,96 (113871,92, 203556,95)	291,64 (213,25, 381,21)	1,73 (-1,71, 5,3)
Romania	6474,54 (4624,84, 8535,81)	145,61 (104,01, 191,97)	8512,71 (5761,1, 11654,25)	230,81 (156,2, 315,99)	0,43 (-2,86, 3,84)
Russia	33968,75 (24553,36, 44269,29)	145,19 (104,94, 189,21)	60980,8 (43118,84, 81494,72)	317 (224,14, 423,63)	1,26 (-2,22, 4,85)
Rwanda	205171,64 (155906,09, 263711,51)	135,85 (103,23, 174,62)	318515,34 (244975,48, 410474,84)	217,09 (166,97, 279,77)	1,31 (-2,17, 4,93)
Saint Kitts and Nevis	5896,6 (3738,19, 8173,41)	82,21 (52,12, 113,95)	9109,38 (5872,57, 12939,7)	71,79 (46,28, 101,98)	5,03 (1,61, 8,56)
Saint Lucia	211,74 (165,09, 258,38)	512,04 (399,23, 624,82)	367,04 (274,58, 469,23)	616,79 (461,42, 788,51)	1,63 (-1,49, 4,86)
Saint Vincent and the Grenadines	526,54 (392,59, 665,18)	383,21 (285,72, 484,11)	1208,93 (880,71, 1604,63)	692,3 (504,34, 918,9)	-0,19 (-2,98, 2,69)
Samoa	409,02 (309,2, 508,68)	371,63 (280,94, 462,18)	700,99 (494,94, 936,47)	619,56 (437,44, 827,68)	1,04 (-2,02, 4,19)
San Marino	426,75 (300,77, 577,97)	260,79 (183,81, 353,2)	975,35 (697,37, 1292,31)	461,48 (329,95, 611,44)	-2,1 (-4,9, 0,79)
Sao Tome and Principe	35,1 (25,64, 46,28)	148,99 (108,84, 196,48)	90,89 (64,17, 122,55)	274,6 (193,88, 370,23)	-1,13 (-3,91, 1,72)
Saudi Arabia	63,3 (44,06, 85,19)	52,08 (36,25, 70,09)	130,61 (88,02, 178,38)	63,59 (42,85, 86,85)	4,8 (1,66, 8,04)
Senegal	11845,34 (7647,46, 16401,95)	73,83 (47,66, 102,22)	60143,98 (40256,03, 84912,98)	168,32 (112,66, 237,64)	0,6 (-2,29, 3,58)
Serbia	8568,37 (6491,34, 10819,11)	112,44 (85,18, 141,97)	24717,72 (18007,01, 32152,48)	163,33 (118,98, 212,45)	2,81 (-0,22, 5,92)
Seychelles	24698,83 (17635,05, 32343,31)	262,82 (187,66, 344,17)	39390,67 (27662,5, 52728,49)	450,34 (316,26, 602,83)	-0,25 (-3,34, 2,94)
Sierra Leone	79,91 (56,99, 105,54)	109,42 (78,03, 144,52)	294,73 (202,36, 406)	288,54 (198,11, 397,47)	0,78 (-2,11, 3,76)
Singapore	3093,06 (2367,37, 3888,83)	84,69 (64,82, 106,48)	8286,03 (6085,9, 10907,72)	100,02 (73,46, 131,66)	2,61 (-0,68, 6,01)
Slovakia	4771,14 (3417,17, 6279,57)	156,57 (112,14, 206,08)	10547,83 (6710,8, 15099,32)	186,11 (118,41, 266,42)	0,94 (-2,73, 4,74)
Slovenia	10916,45 (8215,64, 13809,35)	206,64 (155,51, 261,4)	18205,2 (13024,48, 24399,4)	334,83 (239,54, 448,75)	1,72 (-1,69, 5,25)
Solomon Islands	4415,54 (3166,89, 5895,2)	224,02 (160,67, 299,08)	7566,29 (5251,44, 10288,09)	364,77 (253,17, 495,99)	0,57 (-2,58, 3,83)

Somalia	884,11 (604,01, 1261,14)	259,69 (177,42, 370,44)	3352,41 (2335,85, 4499,65)	511,32 (356,27, 686,31)	2,51 (-0,96, 6,1)
South Africa	9553,18 (6696,97, 12721,26)	133,66 (93,7, 177,98)	25327,18 (18430, 33490,71)	124,5 (90,6, 164,63)	0,57 (-1,83, 3,03)
South Korea	70959,77 (55684,92, 86291,44)	192,67 (151,2, 234,3)	235095,89 (187437,87, 282619,18)	422,92 (337,19, 508,41)	0,76 (-2,27, 3,88)
South Sudan	4437,16 (3013,89, 6151,2)	75,73 (51,44, 104,98)	7579,41 (4993,75, 10473,54)	81,65 (53,79, 112,83)	0,79 (-2,17, 3,84)
Spain	119288,41 (91186,54, 153440,72)	307,59 (235,13, 395,66)	178193,06 (126980,46, 243735,89)	387,2 (275,92, 529,62)	0,37 (-2,22, 3,02)
Sri Lanka	18900,01 (13764,01, 24415,93)	109,75 (79,92, 141,78)	89976,43 (64069,11, 119466,28)	411,71 (293,16, 546,65)	0,31 (-2,18, 2,87)
Sudan	12832,76 (8858,02, 17463,74)	63,53 (43,85, 86,46)	42670,25 (29232,54, 58476,8)	104,56 (71,63, 143,3)	1,4 (-2,15, 5,09)
Suriname	825,63 (599,05, 1068,78)	213,56 (154,95, 276,45)	2686,22 (1874,94, 3563,35)	466,45 (325,57, 618,76)	-2,31 (-5,12, 0,58)
Sweden	17771,3 (13669,65, 22376,55)	206,91 (159,16, 260,53)	29323,47 (21890,94, 37887,34)	286,85 (214,14, 370,63)	-0,93 (-4,56, 2,84)
Switzerland	16465,75 (12701,6, 20434,57)	239,84 (185,01, 297,65)	25066,5 (18065,8, 33662,78)	285,65 (205,87, 383,61)	2,96 (-0,33, 6,34)
Syria	8344,55 (5601,44, 11156,59)	64,71 (43,44, 86,52)	23445,56 (15571,92, 32303,86)	161,79 (107,46, 222,92)	3,19 (-0,5, 7,02)
Taiwan (Province of China)	38839,16 (29219,53, 49715,66)	190,4 (143,24, 243,71)	98560,07 (71126,34, 128951,3)	417,27 (301,12, 545,94)	-1,31 (-4,16, 1,63)
Tajikistan	5230,42 (4037,51, 6460,95)	97,3 (75,11, 120,19)	23099,34 (17247,83, 29053,73)	243,35 (181,7, 306,07)	3,2 (-0,08, 6,59)
Tanzania	61183,43 (43834,15, 79332,15)	107,57 (77,07, 139,48)	153519,36 (103740,45, 214285,3)	218,96 (147,96, 305,63)	-0,27 (-3,54, 3,11)
Thailand	516,88 (376,62, 687,03)	66,02 (48,1, 87,75)	1893,84 (1315,38, 2518,85)	141,88 (98,54, 188,7)	1,19 (-2,08, 4,56)
Timor-Leste	2248,18 (1723,37, 2800,19)	61,38 (47,05, 76,45)	8505,33 (6304,52, 11268,5)	107,37 (79,59, 142,25)	1,25 (-1,19, 3,74)
Togo	6,55 (4,58, 8,83)	388,29 (271,28, 523,39)	8,44 (5,77, 11,49)	597,99 (408,64, 814,61)	-0,44 (-3,35, 2,55)
Tokelau	307,3 (223,35, 395,83)	317,5 (230,76, 408,96)	522,27 (368,07, 697,37)	510,28 (359,62, 681,35)	1,42 (-1,65, 4,6)
Tonga	7620,26 (5866,75, 9264,15)	633,43 (487,67, 770,08)	14307,37 (10083,87, 18872,97)	1031,19 (726,79, 1360,26)	-2,03 (-5,48, 1,55)
Trinidad and Tobago	6165,98 (4045,03, 8659,14)	73,06 (47,93, 102,61)	23879,67 (15241,18, 33755,42)	206,36 (131,71, 291,71)	-0,43 (-3,63, 2,88)
Tunisia	79270,95 (55316,03, 104384,84)	132,62 (92,54, 174,64)	153526,6 (106800,75, 205564,69)	188,7 (131,27, 252,66)	1,38 (-1,28, 4,11)

Turkey	3757 (2950,5, 4711,28)	101,4 (79,63, 127,15)	15217,28 (11522,13, 19303,54)	299,37 (226,68, 379,76)	1,11 (-1,89, 4,19)
Turkmenistan	43,56 (31,12, 57,57)	466,06 (333, 615,98)	81,5 (56,44, 113,57)	690,77 (478,41, 962,62)	0,65 (-1,91, 3,28)
Tuvalu	9477,48 (6034,85, 13269,01)	54,73 (34,85, 76,63)	26300,98 (16928,62, 36842,37)	63,96 (41,17, 89,6)	0,03 (-2,87, 3,02)
Uganda	68794,9 (50593,94, 89852,6)	130,63 (96,07, 170,62)	76456,51 (54113,54, 103887)	173,6 (122,87, 235,88)	-0,57 (-3,23, 2,17)
UK	2126,19 (1589,39, 2751,48)	113,57 (84,9, 146,98)	19465,05 (12913,06, 27862,42)	210,62 (139,73, 301,49)	1 (-2,01, 4,1)
Ukraine	128306,34 (98566,37, 163561,07)	223,25 (171,5, 284,59)	247992,1 (172190,89, 337083,69)	368,92 (256,16, 501,46)	1,56 (-1,89, 5,13)
United Arab Emirates	19718,28 (14433, 25179,59)	76,13 (55,72, 97,21)	44792,16 (31154,72, 60311,35)	78,95 (54,91, 106,3)	0,7 (-2,83, 4,36)
Uruguay	734879,44 (583451,11, 914525,7)	289,77 (230,06, 360,61)	1631916,93 (1261918,53, 2106610,88)	497,57 (384,76, 642,3)	0,6 (-1,75, 3)
USA	241,33 (172,43, 315,28)	227,64 (162,65, 297,4)	591,05 (412,55, 790,77)	568,39 (396,74, 760,46)	3,61 (0,39, 6,94)
Uzbekistan	6318,67 (5301,88, 7295,64)	201,27 (168,88, 232,39)	10891,79 (8514,98, 13682,46)	316,98 (247,81, 398,19)	-0,97 (-4,06, 2,21)
Vanuatu	17965,05 (13978,32, 22231,09)	85,76 (66,73, 106,13)	109606,39 (83993,92, 138606,39)	325,46 (249,41, 411,57)	1,62 (-1,2, 4,53)
Venezuela	228,51 (154,4, 331,01)	150,95 (101,99, 218,66)	1062,08 (732,65, 1459,64)	360,58 (248,74, 495,55)	2,14 (-1,02, 5,41)
Vietnam	28011,28 (20579,48, 36286,43)	148,76 (109,29, 192,71)	107103,62 (76415,06, 142405,85)	381,57 (272,24, 507,34)	2,19 (-0,7, 5,17)
Virgin Islands US	85201 (63148,85, 108802,14)	125,4 (92,95, 160,14)	224625,08 (161343,92, 297491,37)	233,08 (167,42, 308,69)	0,59 (-2,5, 3,79)
Yemen	6705,94 (4635,31, 9171,95)	48,84 (33,76, 66,81)	27616,42 (19299,95, 37875,8)	87,66 (61,26, 120,23)	1,66 (-1,37, 4,77)
Zambia	8290,96 (6253,28, 10402,65)	104,38 (78,73, 130,97)	21066,2 (15555,42, 27319,96)	115,51 (85,29, 149,8)	1,31 (-1,99, 4,71)
Zimbabwe	9941,29 (7637,95, 12352,45)	96,16 (73,88, 119,48)	29159,02 (20774,23, 37966,79)	194,25 (138,39, 252,93)	0,12 (-2,59, 2,9)

No., number; DALYs, disability-adjusted life years; ASDR, age-standardized DALY rate; UI, uncertainty interval; EAPC, estimated annual percentage change; CI, confidence interval.

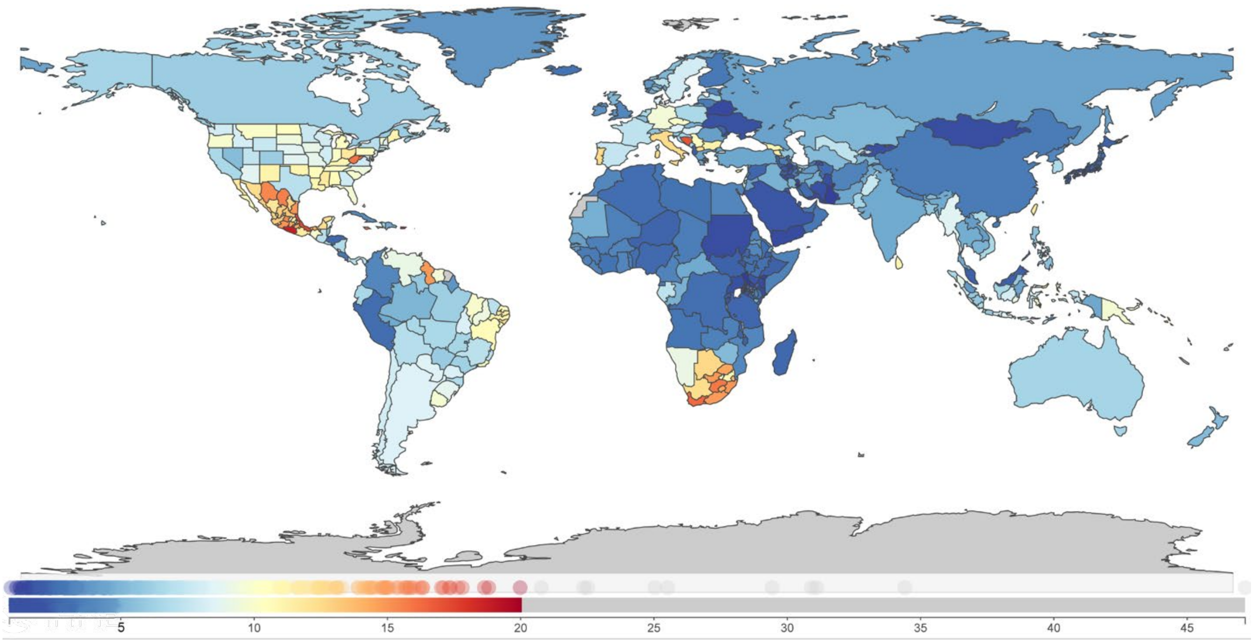

Figure S1. ASMR for T2DM attributable to dietary risk factors in 2019 in 204 countries and territories (Source: Institute for Health Metrics and Evaluation (IHME). GBD Compare. Seattle, WA: IHME, University of Washington, 2019. Available from https://vizhub.healthdata.org/gbd-compare)

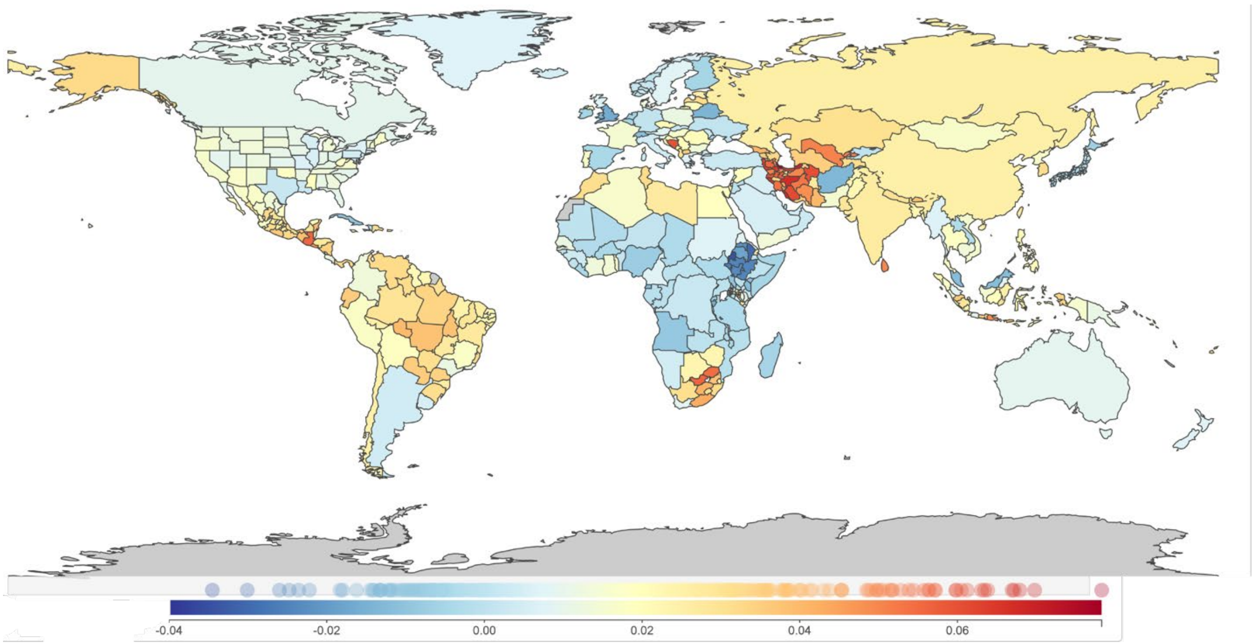

Figure S2. EAPC of ASMR for T2DM attributable to dietary risk factors between 1990 and 2019 in 204 countries and territories (Source: Institute for Health Metrics and Evaluation (IHME). GBD Compare. Seattle, WA: IHME, University of Washington, 2019. Available from https://vizhub.healthdata.org/gbd-compare)

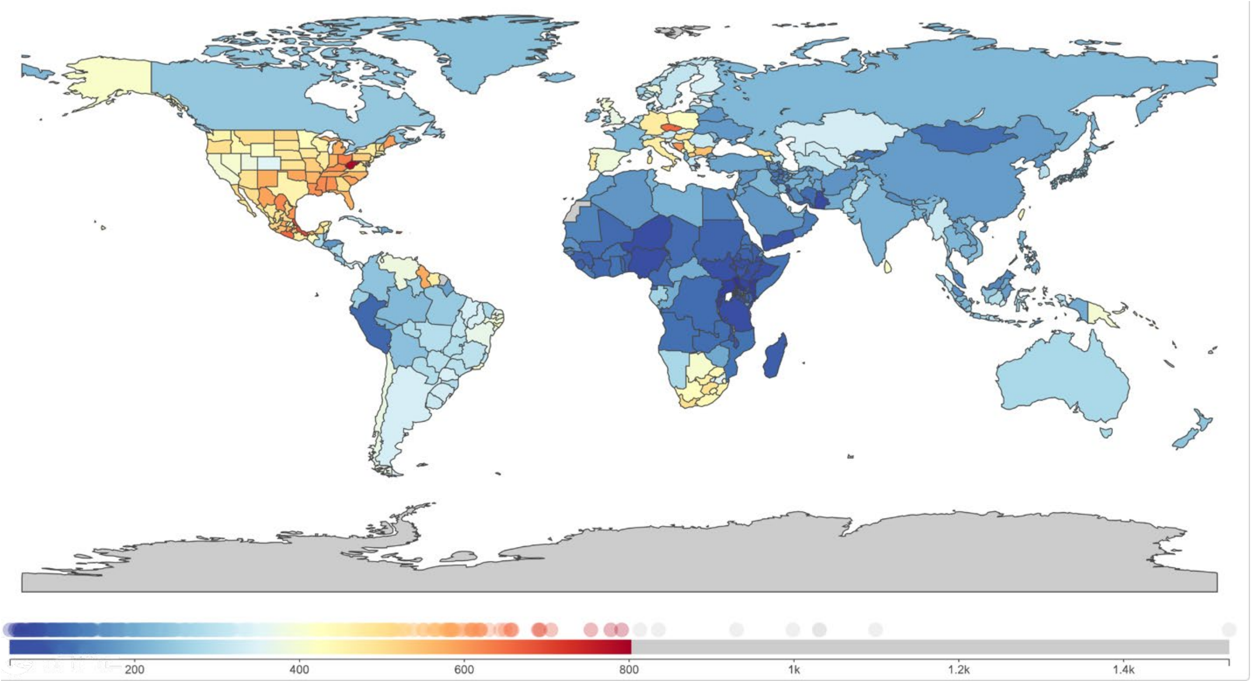

Figure S3. ASDR for T2DM attributable to dietary risk factors in 2019 in 204 countries and territories (Source: Institute for Health Metrics and Evaluation (IHME). GBD Compare. Seattle, WA: IHME, University of Washington, 2019. Available from https://vizhub.healthdata.org/gbd-compare)

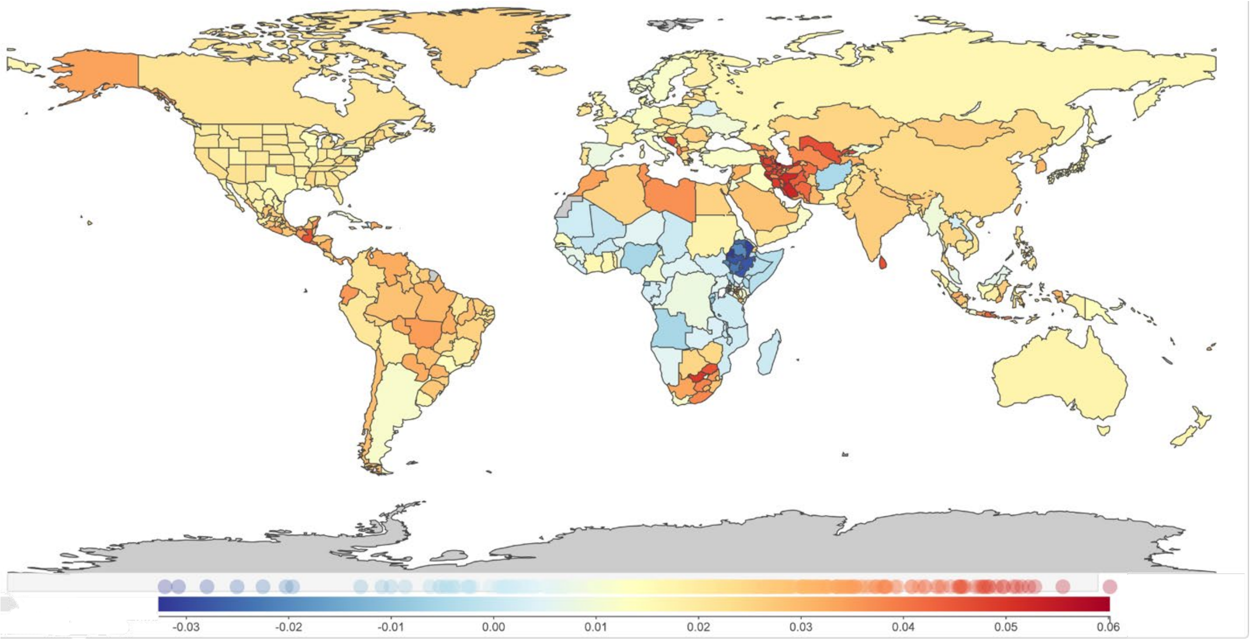

Figure S4. EAPC of ASDR for T2DM attributable to dietary risk factors, between 1990 and 2019 in 204 countries and territories (Source: Institute for Health Metrics and Evaluation (IHME). GBD Compare. Seattle, WA: IHME, University of Washington, 2019. Available from https://vizhub.healthdata.org/gbd-compare)
